# Accommodating Ontologies to Biological Reality—Top-Level Categories of Cumulative-Constitutively Organized Material Entities

**DOI:** 10.1371/journal.pone.0030004

**Published:** 2012-01-09

**Authors:** Lars Vogt, Peter Grobe, Björn Quast, Thomas Bartolomaeus

**Affiliations:** 1 Institut für Evolutionsbiologie und Ökologie, Universität Bonn, Bonn, Germany; 2 Forschungsmuseum Alexander Koenig Bonn, Bonn, Germany; University of South Florida College of Medicine, United States of America

## Abstract

**Background:**

The Basic Formal Ontology (BFO) is a top-level formal foundational ontology for the biomedical domain. It has been developed with the purpose to serve as an ontologically consistent template for top-level categories of application oriented and *domain reference ontologies* within the Open Biological and Biomedical Ontologies Foundry (OBO). BFO is important for enabling OBO ontologies to facilitate in reliably communicating and managing data and metadata within and across biomedical databases. Following its intended single inheritance policy, BFO's three top-level categories of material entity (i.e. ‘object’, ‘fiat object part’, ‘object aggregate’) must be exhaustive and mutually disjoint. We have shown elsewhere that for accommodating all types of constitutively organized material entities, BFO must be extended by additional categories of material entity.

**Methodology/Principal Findings:**

Unfortunately, most biomedical material entities are cumulative-constitutively organized. We show that even the extended BFO does not exhaustively cover cumulative-constitutively organized material entities. We provide examples from biology and everyday life that demonstrate the necessity for ‘portion of matter’ as another material building block. This implies the necessity for further extending BFO by ‘portion of matter’ as well as three additional categories that possess portions of matter as aggregate components. These extensions are necessary if the basic assumption that all parts that share the same granularity level exhaustively sum to the whole should also apply to cumulative-constitutively organized material entities. By suggesting a notion *of granular representation* we provide a way to maintain the single inheritance principle when dealing with cumulative-constitutively organized material entities.

**Conclusions/Significance:**

We suggest to extend BFO to incorporate additional categories of material entity and to rearrange its top-level material entity taxonomy. With these additions and the notion of *granular representation*, BFO would exhaustively cover all top-level types of material entities that application oriented ontologies may use as templates, while still maintaining the single inheritance principle.

## Introduction

The importance of databases in biomedical sciences constantly increases and with it the importance of organizing and standardizing their contents. Bio-ontologies play an important role in this standardization process (e.g. [1]–[4]), because they can provide controlled vocabularies with explicit definitions and unambiguous designations in a highly formalized syntax and a standardized format (e.g. web ontology language, OWL). This provides a framework for an extensible data organization. As a consequence, the interest in bio-ontology research increased significantly within the last decade.

Many ontologies are developed with a specific application in mind. Such *terminology-based application ontologies* [5], [6], like the Gene Ontology (GO) or the Systematized Nomenclature of Medicine-Clinical Terms (SNOMED-CT), aim to provide a system of terms (i.e. controlled vocabulary), which meets particular purposes and needs of a specific research discipline. As a consequence, respective developers often focus exclusively on definitions of very specific types of entities, and not much attention is paid to top-level categories of the ontology. This can lead to ontological inconsistencies within the ontology and incompatibilities with other ontologies, which, at its turn, would also affect the comparability and compatibility of the contents of databases that use these ontologies. *Domain reference ontologies* [5], [6], like the Foundational Model of Anatomy (FMA), represent general-purpose resources that are developed to support a range of different types of research for a specific domain. They have the potential to provide a template of top-level categories for application ontologies of their domain, resulting in an increased comparability and compatibility of respective application ontologies. However, domain reference ontologies cannot provide such a standard across domains. This is the purpose of *formal top-level ontologies* [5], [6], as for instance the Descriptive Ontology for Linguistic and Cognitive Engineering (DOLCE) or the Basic Formal Ontology (BFO), which provide domain-independent theories within a formal framework of axioms and definitions for most general terms and concepts. The idea is that if domain reference ontologies make use of the machinery of a formal top-level ontology and apply its structure to their specific domain of reality, the comparability and compatibility across different domain reference ontologies will increase (e.g. [6]–[9]). Therefore, formal top-level ontologies take in a central role in the standardization of ontologies and thus in the standardization of scientific database contents.

The Open Biological and Biomedical Ontologies Foundry (OBO; http://www.obofoundry.org) provides a central platform that facilitates in standardizing ontologies and in establishing best practices in ontology development for the biomedical domain. It represents one of the most important resources and repositories for biomedical ontologies. Within the OBO Foundry, the Basic Formal Ontology (BFO; http://www.ifomis.org/bfo; [10]), which has been developed as a realist ontology (i.e. representing kinds of entities and their divisions that exist in the mind-independent world), serves as a formal top-level ontology. It provides the top-level hierarchical structure that serves as a template for the development of various application and domain reference ontologies within the OBO Foundry. In other words, for all accepted ontologies of the OBO Foundry applies that cross-ontological comparability of application and domain reference ontologies depends on BFO.

An increasing number of ontologies are being developed that use BFO for their top-level framework (http://www.ifomis.org/bfo/users). Because it has been developed with the primary intention to be used in the structuring of biomedical domain ontologies [11], it does not contain physical, chemical, biological, medical or other terms that properly fall within these special science domains. These terms must be defined in the respective domain reference ontologies.

One of the important design principles that has been used for developing BFO is the *single inheritance policy*, which requires all defined categories to be disjoint and exhaustive – categories must be mutually exclusive relative to a given level of granularity [11]. As a consequence, each BFO category has exactly one single asserted parent class (except for the root category). Single inheritance represents an important design principle because multiple inheritance often goes hand in hand with errors in ontology construction (e.g. [12]) and often substantially complicates or even prohibits coherent integration across ontologies (e.g. [13]). Single inheritance, on the other hand, supports clear statements of definitions, easier and more reliable ontology curation, and it allows the use of more powerful reasoning tools and a single measure of distance between two classes (e.g. [14]).

From the application of the single inheritance principle to the three different sub-categories of material entity that BFO distinguishes follows that any given particular material entity must instantiate exactly one of the following three types of material entity for any given level of granularity: ‘fiat object part’, ‘object’, or ‘object aggregate’ (for definitions see Table 1). In other words, whereas a given material entity, as for instance an individual human being, is an object at one level of granularity (e.g. human body), it may at the same time be an object aggregate at a finer level of granularity (e.g. the aggregate of all cells of the individual human being), so that it cannot be both at a given level of granularity. Therefore, if BFO wants to live up to its role as the provider of a formal top-level ontology for scientific biomedical ontologies, its top-level categories of material entity must be exhaustive and mutually disjoint within the same level of granularity.

**Table 1:** Definitions of the Basic Types of Material Entity of the Basic Formal Ontology (BFO Version 1.1).

Definition	Parent Class Affiliation	Link/ID
**‘material entity’:** *“An independent continuant that is spatially extended whose identity is independent of that of other entities and can be maintained through time. Note: Material entity subsumes object, fiat object part, and object aggregate, which assume a three level theory of granularity, which is inadequate for some domains, such as biology. Examples: collection of random bacteria, a chair, dorsal surface of the body”*	‘independent continuant’	http://www.ifomis.org/bfo/1.1/snap#MaterialEntity
**‘object’:** *“A material entity that is spatially extended, maximally self-connected and self-contained (the parts of a substance are not separated from each other by spatial gaps) and possesses an internal unity. The identity of substantial object entities is independent of that of other entities and can be maintained through time. Examples: an organism, a heart, a chair, a lung, an apple”*	‘material entity’	http://www.ifomis.org/bfo/1.1/snap#Object
**‘fiat object part’:** *“A material entity that is part of an object but is not demarcated by any physical discontinuities. Examples: upper and lower lobes of the left lung, the dorsal and ventral surfaces of the body, the east side of Saarbruecken, the lower right portion of a human torso”*	‘material entity’	http://www.ifomis.org/bfo/1.1/snap#FiatObjectPart
**‘object aggregate’:** *“A material entity that is a mereological sum of separate object entities and possesses non-connected boundaries. Examples: a heap of stones, a group of commuters on the subway, a collection of random bacteria, a flock of geese, the patients in a hospital”*	‘material entity’	http://www.ifomis.org/bfo/1.1/snap#ObjectAggregate

In the following we focus on top-level categories of material entity and their spatio-structural properties. We systematically evaluate and assess whether BFO's three categories of material entity really exhaustively cover all types of material entity that are relevant to the biomedical domain. Elsewhere we have already shown that this is not the case for *constitutively* organized material entities and we suggested extending BFO by adding further top-level categories of material entity [4]. These necessary extensions to BFO are briefly summarized in the first part of this paper. In the second part we evaluate and assess whether this extended BFO also exhaustively covers all types of *cumulative-constitutively* organized material entities. This is important insofar, as most biomedical material entities are cumulative-constitutively organized. By referring to adequate examples from biology we demonstrate the necessity of further extending BFO with additional top-level categories, which we introduce and discuss. We argue that this extension is necessary if the basic assumption that all parts sharing the same granularity level exhaustively sum to the whole should also apply to cumulative-constitutively organized material entities. By suggesting a notion of granular representation we provide a way to maintain the single inheritance principle also when dealing with cumulative-constitutively organized material entities. We conclude by making suggestions for how all top-level categories of material entity of the extended BFO can be best subsumed under a top-level taxonomy that accommodates all types of constitutively and cumulative-constitutively organized material entities.

## Results

### Top-Level Categories of Constitutively Organized Material Entities

#### Constitutive Granularity

According to BFO's definitions of ‘object aggregate’ and ‘fiat object part’, an object aggregate consists of objects and an object consists of fiat object parts. This implies a very simple granularity scheme consisting of three levels of granularity (in the following we use ‘<’ to indicate part-of or lower-level-than relationships of a very general notion of granularity that is based on proper direct parthood relations): *fiat object part<object<object aggregate*.

**Figure 1:**
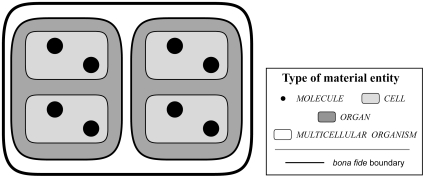
Constitutive Granularity. A *constitutive granularity* of molecules, cells and organs of a multicellular organism. It is characteristic of constitutive granularities that all objects belonging to one level of granularity are parts of objects of the next coarser level of granularity: all molecules are part of cells, all cells part of organs, and all organs part of multicellular organisms. Moreover, the sum of all objects of one level of a constitutive granularity yields the maximal object, which is in this case a multicellular organism.

This very simple scheme represents, of course, an oversimplification of reality. The scheme becomes more realistic and also more complicated when allowing for a more complex organization of reality, with objects of finer levels of granularity constituting objects at coarser levels. Like for instance atoms constituting a molecule and molecules constituting a cell. In other words, aggregates of objects of a finer level of granularity constitute *bona fide* objects at coarser granularity, resulting in a *constitutive hierarchical organization* of *bona fide* objects of different granularity that are nested within one another (see Fig. 1) (for *constitutive hierarchy* see [15], [16]).

Most granularity schemes suggested in the literature so far presuppose such a constitutive organization of material entities (e.g. [17], [18] for an exception see [19]). They assume that: higher level entities consist of physically joined elements, all objects belonging to one level of granularity form parts of objects of the next higher level of granularity, summing together all objects that belong to one level of granularity yields a maximal *bona fide* object – all parts that share the same granularity level exhaustively sum to the whole (e.g., the sum of all cells of a human body yields the human body as a whole).

Constitutive organization results in a granularity scheme consisting of several blocks of the simple three-leveled granularity scheme described above. Each block forms a level of granularity that consists of three sublevels. Each distinguishable level of granularity of *bona fide* objects has an associated level of fiat object parts and an associated level of object aggregates (each block demarcated by square brackets): [*fiat atom part*<*atom*<*atom aggregate*]<[*fiat molecule part*<*molecule*<*molecule aggregate*]<[*fiat cell part*<*cell*<*cell aggregate*]<[*fiat organ part*<*organ*<*organ aggregate*]<[*fiat body part*<*body*<*body aggregate*].

The granularity relations within each three-leveled block and those between different *bona fide* object levels can be determined universally. Determining the other granularity relations, as for instance between atom aggregates and fiat molecule parts, is not that straight forward and must be decided on a case by case basis (see [4], [19]).

#### Implications from a Constitutive Granularity: Cross-Granular Multiple Instantiations and the Single Inheritance Principle

According to the *single inheritance principle*, any particular entity belongs to only one of BFO's basic categories of material entity: it is either an object aggregate, an object, or a fiat object part, and it cannot instantiate more than one of these basic categories. In other words, each class has maximally one single asserted direct parent class within BFO's hierarchical taxonomy of top-level categories [11]. It is possible to adhere to this policy as long as one only considers the relations between fiat object parts, objects, and object aggregates belonging to the same general granularity level (e.g. fiat cell parts, cells, and cell aggregates). However, as soon as one accounts for the granular nature of the spatio-structural organization of material entities, it is generally asserted that multiple inheritance cannot be avoided [11].

**Figure 2:**
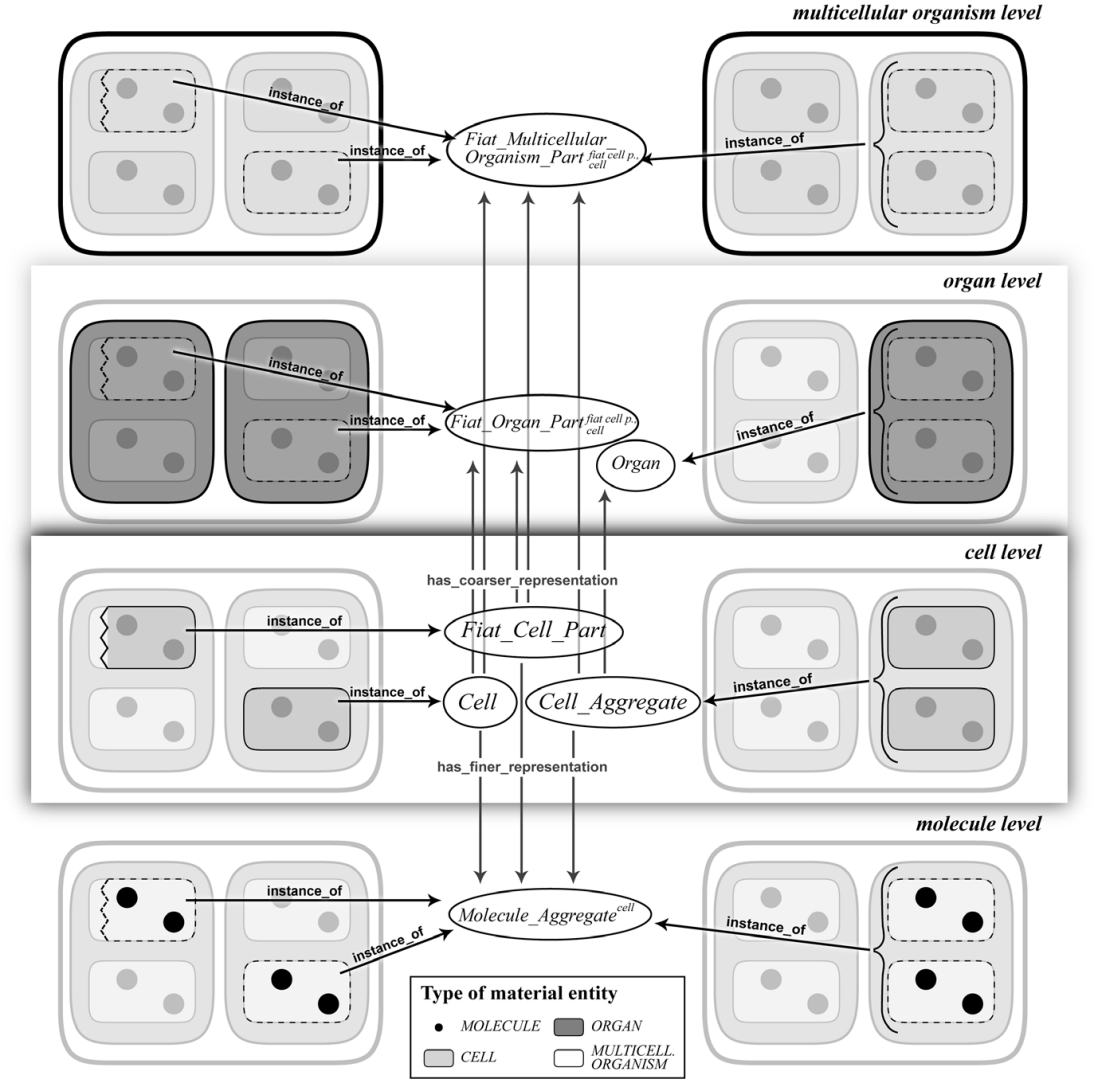
Granular Representation of a Particular Fiat Cell Part, Cell, and Cell Aggregate. At the cellular level a particular fiat cell part instantiates the ‘fiat cell part’ category, which is a subcategory of ‘fiat object part’. A particular cell instantiates the ‘cell’ category, which is a subcategory of ‘object’. A particular cell aggregate instantiates the ‘cell aggregate’ category, which is a subcategory of ‘object aggregate’. At the finer molecular level, they all instantiate different subcategories of ‘molecule aggregate’ (i.e. subcategories of ‘object aggregate’). At the coarser organ and multicellular organism level they instantiate subcategories of ‘fiat organ part’ and ‘fiat multicellular organism part’ (i.e. subcategories of ‘fiat object part’). In some cases a particular cell aggregate instantiates a subcategory of ‘organ’ (i.e. subcategory of ‘object’). Although each particular entity instantiates multiple categories, these categories do not necessarily have to stand in a class-subclass relation to one another. Instead, they are different *granular representations* (see *2.2.3 Granular Representation and the Single Inheritance Principle*) of the same specific type of material entity (i.e. *FIAT CELL PART*, *CELL*, *CELL AGGREGATE*).

The reason for this conclusion is the fact that any particular material entity instantiates several supposedly disjoint classes. For instance, every instance of the type *CELL* (from here onward, we will use italic upper case for designating types or kinds of material entity, whereas we use single parenthesis (‘…’) for designating categories/classes within an ontology) not only instantiates the class ‘cell’, which would have to be classified as a subcategory of BFO's category ‘object’. Because every cell, due to its granular nature, is composed of aggregated molecules, every instance of *CELL* also instantiates a specific subcategory of ‘molecule aggregate’, which would have to be classified as a subcategory of BFO's ‘object aggregate’. Moreover, since molecules are composed of aggregated atoms, every cell instantiates a specific subcategory of ‘atom aggregate’ as well. In case the cell belongs to a multicellular organism, it also instantiates the category ‘fiat multicellular organism part’, which would have to be classified as a subcategory of BFO's ‘fiat object part’. The cell may even instantiate the categories ‘fiat tissue part’ and ‘fiat organ part’ in case it is part of a particular tissue that is part of a particular organ (Fig. 2). These *multiple cross-granular instantiations* are not restricted to object entities but hold for all particular material entities – independent of whether they are constitutively or cumulative-constitutively organized. They all not only instantiate an ‘object’ category but also ‘object aggregate’ categories at finer levels of granularity and ‘fiat object part’ categories at coarser levels (except they are the finest or coarsest object entities within a given granular organization).

This interpretation implies that, from a granular perspective, any type of material entity has more than one parent class and therefore exhibits multiple inheritance, which violates the single inheritance policy of BFO. This is why mutual exclusiveness of a given category is considered to be relative to a given level of granularity [11], and any application or domain reference ontology is considered to be necessarily restricted to a particular level of granularity, if it wants to adhere to that policy. If one wants to refer to a specific type of *CELL* at a specific level of granularity one would thus use the respective category of this specific level of granularity from an ontology that is restricted to this specific level of granularity and the single inheritance principle would be satisfied. As a consequence, all relations between entities from different levels of granularity would then have to be dealt with using *cross-ontology relations*. As long as one is only dealing with constitutively organized material entities, this approach of dealing with *cross-granular multiple instantiation* is not only reasonable but also practicable.

#### Extending the Basic Formal Ontology

**Figure 3:**
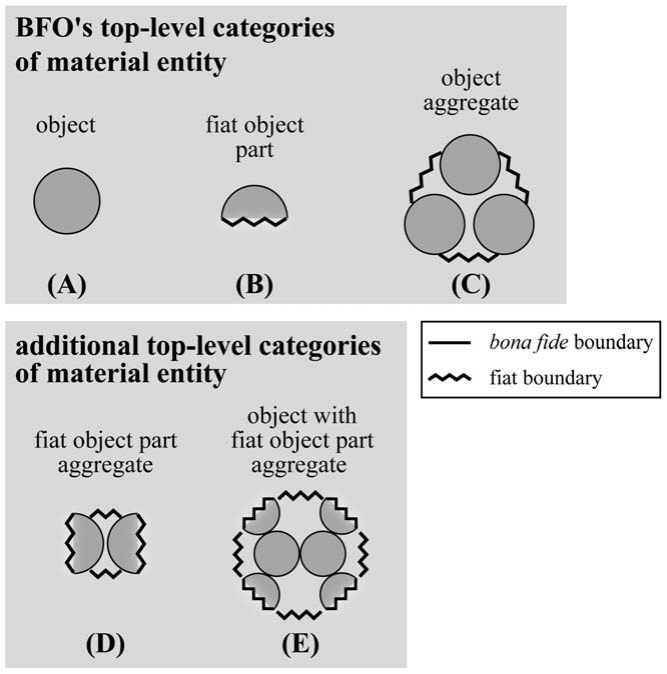
First Order Categories of Material Entity. A) –C) The three different top-level categories of material entity that the Basic Formal Ontology currently distinguishes (BFO, version 1.1). D) & E) Two additional top-level categories of material entity that are currently not recognized by BFO. With the exception of ‘object’, all categories possess some fiat boundary and thus are fiat wholes. (modified from [4]).

**Figure 4:**
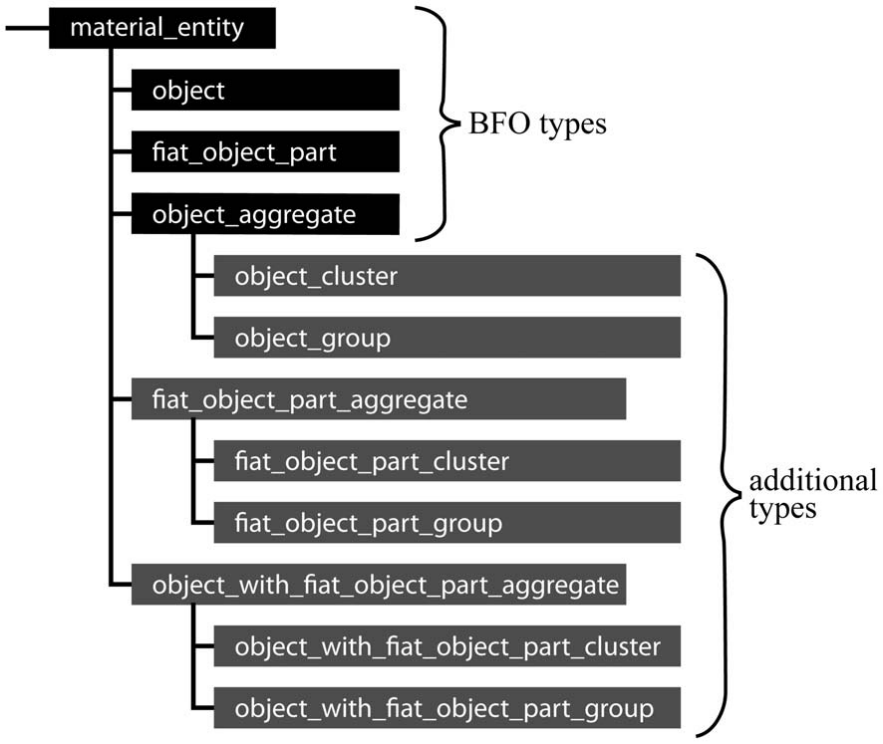
Taxonomy of Top-Level Categories of Constitutively Organized Material Entities. A taxonomy of top-level categories of material entity and important subcategories that can be distinguished in constitutively organized material entities. *Black boxes indicate Basic Formal Ontology (BFO) categories of material entity and dark grey boxes the additional categories suggested by* [4]. (modified from [4]).

In order to live up to its role as providing a general template for the top-level hierarchical structure of various application and domain reference ontologies for the biomedical domain, BFO's categories of material entity must exhaustively cover all possible types of material entities relevant to the biomedical domain. By systematically evaluating all theoretically possible basic constellations of material building blocks (i.e. constellations of *bona fide* objects and fiat object parts), we have demonstrated that the categories of material entity defined in the current version of BFO (version 1.1; see Table 1) are not exhaustive and also not sufficiently differentiated in order to fulfill this task without causing inconsistencies [4]. With several examples from biology and from everyday life we provided evidence for the necessity of extending BFO's categories of material entity with two additional categories: (i) ‘fiat object part aggregate’ and (ii) ‘object with fiat object part aggregate’ (Figs. 3,4; for definitions see Table 2; [4]).

**Table 2:** Definitions of additional Top-Level Categories of Material Entity for the Basic Formal Ontology (from [4]).

Definition	Parent Class Affiliation
**‘fiat object part aggregate’:** *A material entity that is a mereological sum of separate (i.e. not sharing a fiat boundary with each other) fiat object part entities and possesses non-connected fiat boundaries. Examples: a synapse, the fingers of a hand, a joint, a door hinge, hydrogen bond between molecules, an estuary, mainland of the Russian Federation, mainland of Turkey*	‘material entity’
**‘object with fiat object part aggregate’:** *A material entity that is a mereological sum of separate (i.e. not sharing a fiat boundary with each other) object and fiat object part entities and possesses non-connected boundaries. Examples: a human heart, a power outlet, a train station, a traditional telephone cord connection between two telephones, the territories of Turkey and of England*	‘material entity’
**‘object group’:** *An object aggregate that is a mereological sum of spatially separated object entities, which do not adhere to one another through chemical bonds or physical junctions but, instead, relate to one another merely on grounds of metric proximity. The objects can be separated from one another through space or through other object entities that do not belong to the group. Examples: a heap of stones, a colony of honeybees, the trees of a forest, the fish of a shoal, a group of commuters on the subway, the patients in a hospital*	‘object aggregate’
**‘object cluster’:** *An object aggregate that is a mereological sum of separate object entities, which adhere to one another through chemical bonds or physical junctions that go beyond gravity. Examples: the atoms of a molecule, the molecules forming the membrane of a cell, the epidermis in a human body*	‘object aggregate’
**‘fiat object part group’:** *A fiat object part aggregate that is a mereological sum of spatially separated fiat object part entities, which do not adhere to one another through chemical bonds or physical junctions but, instead, relate to one another merely on grounds of metric proximity. The fiat object parts can be separated from one another through space or through other material entities that do not belong to the group. Examples: the fingers of a hand, a joint, a door hinge, opposite riverside sections, mainland of the Russian Federation*	‘fiat object part aggregate’
**‘fiat object part cluster’:** *A fiat object part aggregate that is a mereological sum of separate fiat object part entities, which adhere to one another through chemical bonds or physical junctions that go beyond gravity. Examples: synapse, hydrogen bond between molecules, an estuary, mainland of Turkey*	‘fiat object part aggregate’
**‘object with fiat object part group’:** *An object with fiat object part aggregate that is a mereological sum of spatially separated object entities and fiat object part entities, which do not adhere to one another through chemical bonds or physical junctions but, instead, relate to one another merely on grounds of metric proximity. The objects and fiat object parts can be separated from one another through space or through other material entities that do not belong to the group. Examples: the equilibrium organ of a lobster, a modern wireless cell phone connection, the territories of Turkey and of England*	‘object with fiat object part aggregate’
**‘object with fiat object part cluster’:** *An object with fiat object part aggregate that is a mereological sum of separate object entities and fiat object part entities, all of which adhere to one another through chemical bonds or physical junctions that go beyond gravity. Examples: a human heart, a power outlet, a train station, a traditional telephone cord connection between two telephones, a polyplacophoran aesthete*	‘object with fiat object part aggregate’

**Figure 5:**
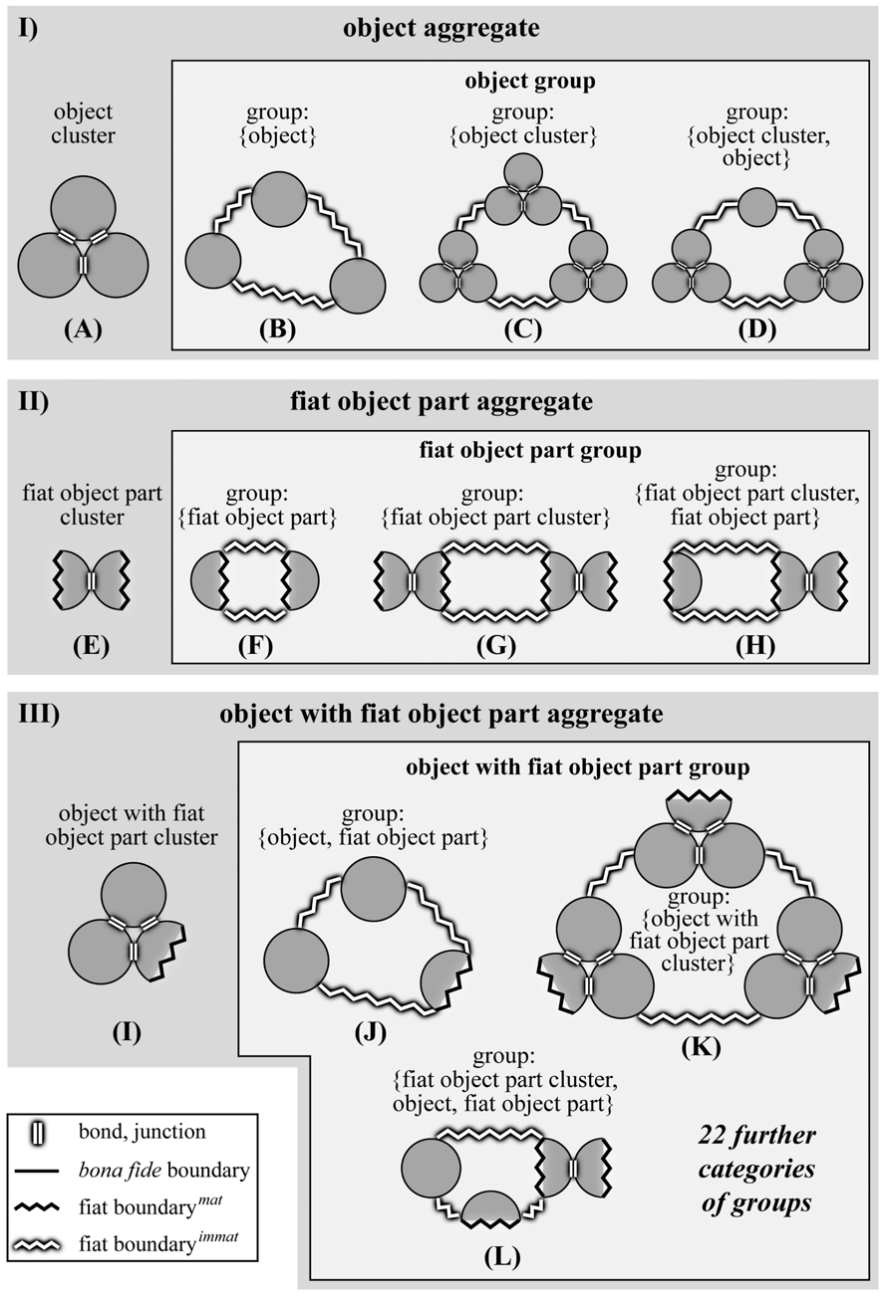
Second Order Categories of Material Entity. Possible subcategories of the three top-level categories of material entity aggregates (I–III) that can be differentiated on grounds of distinguishing two types of relation between the entities of the aggregate (i.e. *metric proximity* and *topological adherence*) and the presence or lack of fiat boundaries*^immat^*: (i) *Clusters* are *not* demarcated by fiat boundaries*^immat^* and are further characterized by topological adherence relations between the entities of the aggregate (through chemical bonds or physical junctions). (ii) *Groups are* demarcated by fiat boundaries*^immat^* and are further characterized by metric proximity relations between the entities of the aggregate – they lack adherence. Since also several clusters can spatially relate to one another on grounds of metric proximity, clusters can be parts of groups as well. A)–D) The four subcategories of ‘object aggregate’ – an ‘object cluster’ and three subcategories of ‘object group’, all of which either consist of objects, object clusters, or both. Note that an object cluster is only demarcated by *bona fide* boundaries and thus does not represent a fiat whole. E)–H) The four subcategories of ‘fiat object part aggregate’ – a ‘fiat object part cluster’ and three subcategories of ‘fiat object part group’, all of which either consist of fiat object parts, fiat object part clusters, or both. I)–K) Four out of 26 subcategories of ‘object with fiat object part aggregate’ – an ‘object with fiat object part cluster’ and three out of 25 possible subcategories of ‘object with fiat object part group’. *Fiat boundary^mat^: demarcates fiat parts of a material entity; fiat boundary^immat^: demarcates fiat parts of an immaterial entity (i.e. a hole*). (modified from [4]).

Moreover, by distinguishing *topological coherence, topological adherence*, and *metric proximity* (Table 3), we have suggested to differentiate between *clusters* and *groups* as two distinct subcategories for each of the three top-level categories of material entity aggregates (Figs. 4,5; for definitions see Table 2; for a detailed discussion see [4]). These additions to BFO provide an exhaustive account of all possible types of material entity and thus eliminate the inconsistencies current application ontologies are dealing with when having to incorrectly classify types of material entity that are currently not covered by BFO to one of its three top-level categories (e.g. [20]).

**Table 3:** Three Foundational Types of Spatio-Topological Relations between Material Entities (from [4]).

Type of material entity	Relation between its parts	Type of inner boundary separating its parts	Characteristics
object or fiat object part	*topological coherence*	fiat boundary^mat^ between fiat object parts	Coherence implies physical continuity and qualitative homogeneity within the object or fiat object part
object cluster	*topological adherence*	bona fide boundary between objects	Adherence implies physical continuity and qualitative heterogeneity within the object cluster
object group	*metric proximity*	bona fide boundary and fiat boundary^immat^ between objects	Metric proximity implies physical separation through spatial gaps between the constitutive objects of the object group

These extensions to BFO are required for realistically modeling material entities that are organized according to a constitutive granularity. When evaluating whether the extended BFO lives up to its intended role of providing a general template for the top-level hierarchical structure of various biomedical application and domain reference ontologies, however, it is also essential to confirm that its underlying granularity scheme is in accordance with biomedical reality. In other words, we must conduct a reality check and evaluate whether biological material entities are really organized according to the presumed constitutive granularity. If this is not the case, we must evaluate the implications it has for applying the single inheritance principle and must investigate whether BFO requires further additions in order to cover also the *cumulative-constitutively* organized biological material entities.

### Cumulative Constitutive Granularity and its Implications regarding the Single Inheritance Principle

Conceptualizing organisms along the lines of their part-whole relations is common practice in biology and results in a hierarchical system of anatomical terms and concepts that is usually referred to as *levels of (biological) organization* or *levels of complexity* in the general biological literature ([21], [22]; see also *scalar hierarchy*, [23], [24]; *cumulative constitutive hierarchy*, [16]; *building block systems*, [25]; *Theorie des Schichtenbaus der Welt*, [26]). The *levels of organization* are comparable to the *levels of granularity* in bioinformatics and ontology literature (e.g., [5], [18], [27]). Several hierarchical systems have been proposed in the past in the biological literature – although not as formally stringent as the granularity schemes proposed in the bioinformatics literature. Some, like Eldredge's somatic hierarchy ([28]; see also [29], [30]) are focused on biological anatomical objects, and usually include ‘subatomic particle’<‘atom’<‘molecule’<‘organelle’<‘cell’<‘tissue’<’organ’<‘organ system’<‘individual organism’ as their typical ranks. Considering the broad consensus among biologists regarding the existence and nature of such basic categories as ‘organism’, ‘organ’, ‘tissue’, ‘cell’, ‘gene’, and ‘molecule’ [31], it is not surprising that the typical levels of granularity distinguished by ontology developers usually include the levels of ‘whole organism’, ‘organ’, ‘tissue/tissue sample’, ‘cell’, ‘subcellular’, and ‘molecular’ [10] and that they thus correlate with the typical levels of organization distinguished by biologists. However, despite these similarities, most biologists would disagree with the implications that the presumption of a constitutive granularity brings about.

#### Cumulative Constitutive Granularity of Biological Material Entities

**Figure 6:**
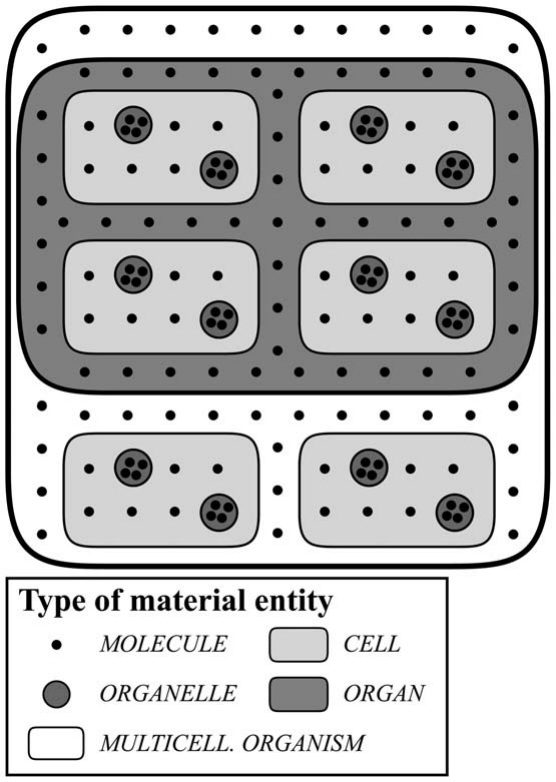
Cumulative Constitutive Granularity. A *cumulative constitutive granularity* of a multicellular organism consisting of molecules, organelles, cells, and organs. It is characteristic of cumulative constitutive granularities that *not all* objects belonging to one granularity level are parts of objects at the next coarser level of granularity – they only have to be part of the maximal whole, which is in this case a multicellular organism. Molecules exist that are not part of cells and cells that are not part of organs. As a consequence, the sum of all objects of one level of a cumulative constitutive granularity does not necessarily yield the maximal object.

Biologists argue that typical biological material entities, like for instance multicellular organisms, are not organized according to a constitutive granularity. Instead, their organization follows a *cumulative constitutive hierarchy* ([16], [32], [33]; see also *somatic hierarchy*, [28]). Contrary to a constitutive granularity, in a cumulative constitutive granularity (Fig. 6) *not all* the objects belonging to one level of granularity necessarily form parts of objects of the next higher level of granularity. This is also in accordance with what we know from reality: not every atom is part of a molecule: e.g. ions, chlorine radicals, noble gases; not every molecule is part of a cell: e.g. extracellular matrix (ECM), which is a macromolecular formation that is a component of tissues and organs and is located outside of cells; not every cell is part of an organ: e.g. erythrocytes, coelomocytes, leukocytes, sperms, egg cells.

As a consequence, and again contrary to a constitutive granularity, all the objects that share the same granularity level *not always* exhaustively sum to the maximal whole (contradicting, e.g., [18], [34]): The totality of all molecules existing in the universe at any given moment in time does not exhaustively sum to the universe as a whole, because there always exist some free atoms and ions that are not part of any molecule; neither does the totality of all atoms exhaustively sum to the universe as a whole, because there always exist free subatomic particles that are not part of any atom; moreover, with respect to most multicellular metazoans (e.g. humans) the totality of cells does not sum to the body as a whole, since that would mean to ignore the extracellular matrix in which the cells are embedded and which provides the topological grid that keeps the cells in their relative position, thereby integrating the cell aggregate into a topological and biological functional whole.

A direct consequence from a cumulative constitutive organization of material entities is that the granularity scheme and the axioms for partitioning material entities are more complicated than they are for constitutively organized material entities (for a theory of granularity for cumulative constitutively organized material entities see [19], which is based on Keet's general formal theory of granularity, [27]).

Surprisingly, although ontology-related research represents a very active field within the biomedical domain and although many biomedical ontologies have been developed (for a list of freely available ontologies see, e.g., NCBO BioPortal, http://bioportal.bioontology.org/, and the Open Biological and Biomedical Ontologies, OBO, http://www.obofoundry.org/), granularity schemes and biomedical ontologies nonetheless commonly presuppose material entities to be universally organized following a constitutive hierarchy (for an exception see [19]).

#### Trans-Granular Multiple Instantiation of the Same Type of Material Entity in a Cumulative Constitutive Granularity

**Figure 7:**
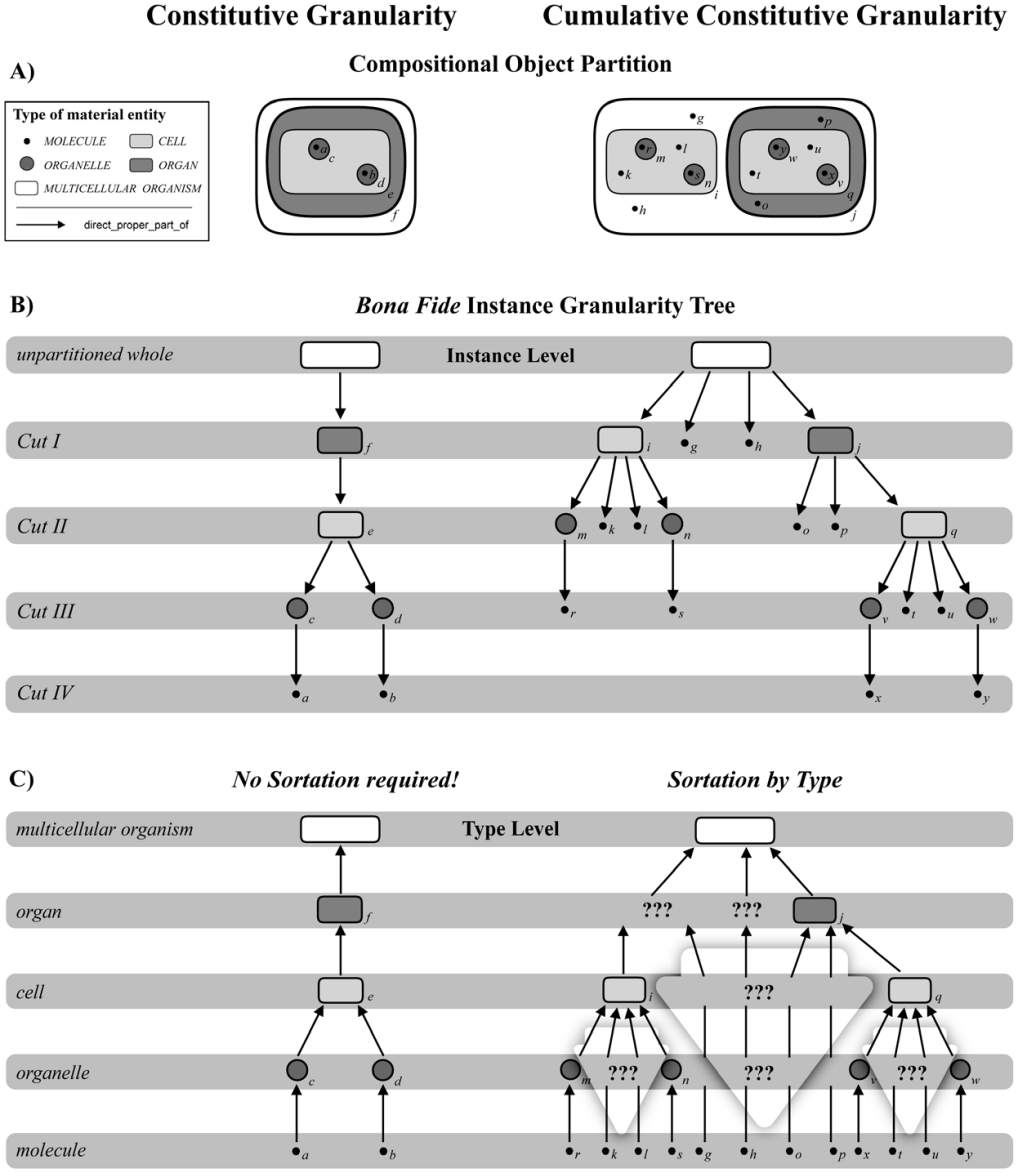
Trans-Granular Multiple Instantiation and the Distinction of Instance Granularity and Type Granularity. A) Left: Compositional partitions of a *constitutively* organized idealized multicellular organism into its constitutive object parts. Four partitions are shown: (i) into organs (*f*); (ii) into organ cells (*e*); (iii) into organelles (*c,d*) of organ cells; and (iv) into organelle molecules (*a,b*). Right: Compositional partitions of a *cumulative-constitutively* organized idealized multicellular organism into its constitutive object parts. The same corresponding four partitions are shown: (i) into organs (*j*) alongside with cells (*i*) and extracellular molecules (*g,h*), both of which are not part of any organ; (ii) into organ cells (*q*) and extracellular molecules (*o,p*) that are part of some organ, organelles (*m*,*n*) that are part of cells which are not part of any organ, and cellular molecules (*k,l*) that are neither part of any organ nor any organelle; (iii) into organelles (*v,w*) of organ cells and molecules (*t,u*) that are part of these organ cells but not part of any organelle, as well as molecules (*r,s*) of organelles of cells which are not part of any organ; and (iv) into organelle molecules (*x,y*) that are part of organ cells. B) Left: The *instance granularity tree* of *constitutively* organized *bona fide* objects based on the corresponding four partitions. Each partition constitutes a *cut* in the instance granularity tree (*Cut I–IV*) and thus an *instance granularity level*. Contrary to cumulative-constitutively organized material entities, particular instances of the same type of material entity do *not* belong to different cuts and thus are restricted to a single level of instance granularity. The types' extensions do *not* transcend the boundaries between instance granularity levels. Right: The *instance granularity tree* of *cumulative-constitutively* organized *bona fide* objects based on the corresponding four partitions. Particular instances of the same type of material entity, like for instance of the type *MOLECULE*, belong to different cuts and thus different levels of the respective instance granularity. In other words, the extension of the type *MOLECULE* transcends the boundaries between instance granularity levels. C) Left: The constitutive instance granularity tree that results from the corresponding four partitions *can* be directly transformed into the respective *type granularity tree* – no sortation required since they are topologically identical. Right: The cumulative constitutive instance granularity tree that results from the corresponding four partitions *cannot* be directly transformed into or mapped upon the respective *type granularity tree*. However, by (i) following the simple and intuitive rule of sortation-by-type (i.e. a type occupies the same granularity level as its finest grained instance) and by (ii) applying a more complicated granularity scheme (Vogt 2010), one can infer a type granularity tree. Unfortunately, this results in types of entities belonging to type granularity levels for which BFO provides no respective categories as templates for granularity specific ontologies (here marked ‘???’). For instance when looking at the cellular level, a cellular ontology that is based on BFO provides no category for molecules that are not part of any cell, since they are neither a cell (i.e. BFO's ‘object’), nor a cell aggregate (i.e. BFO's ‘object aggregate’) or fiat cell part (i.e. BFO's ‘fiat object part’), nor any of the additional categories of the extended BFO. (modified from [19]).

Cumulative-constitutively organized material entities pose problems to ontology design because ontologies are dealing with types (i.e. classes) of material entities. The problems would not exist if ontologies dealt with particular instances or only with constitutively organized material entities. Why this is the case can best be explained with an example: One can partition a multicellular organism into its direct proper objet parts (Fig. 7A). If multicellular organisms were constitutively organized (Fig. 7A left), this compositional object partition would be straight forward. In a first step, the entire organism would be partitioned into its constitutive organ parts. All organs of the multicellular organism would belong to the same *cut* in the corresponding *instance granularity tree* (for cuts see [35], [36]) and thus to the same *instance granularity level*. In a next step, each organ could be further partitioned into its specific direct proper cell parts, forming another cut in the tree and the adjacently finer instance granularity level. As a result, all cells of the multicellular organism would belong to the next finer level of instance granularity. This procedure can be repeated several times, as long as parts of one cut can still be further partitioned into finer object parts (e.g. cells into their organelle parts, organelles into their molecule parts, etc.). All these partitions would result in a set of cuts that constitute an instance granularity tree of particular *bona fide* object entities (Fig. 7B left). All instances belonging to one cut would instantiate the same top-level object type: the first cut would contain all the organs of the multicellular organism, the next cut all its cells, and so on. All object entities of a certain type would belong to the same cut and thus the same instance granularity level.

Therefore, transforming the *instance granularity tree* into a *type granularity tree* would be straight forward: for a given constitutively organized object the instance granularity tree and the type granularity tree are topologically identical (Fig. 7B,C left). As a consequence, the single inheritance principle could be applied to all categories of material entity relative to a given type granularity level, because their extensions do not cross the boundary between different instance granularity levels, and the instance granularity levels correspond with the type granularity levels.

Unfortunately, reality is not that simple. As mentioned above, multicellular organisms are cumulative-constitutively organized (Fig. 7A right). As a consequence, their direct proper object parts are not restricted to objects of the same top-level object type. Partitioning a multicellular organism yields in a first step individual organs surrounded by individual cells that are not part of any organ as well as by individual molecules that are neither part of any organ nor any cell, all of which represent direct proper parts of the multicellular organism (Fig. 7B right). Although these particular parts instantiate different types of material entity, they nevertheless all belong to the same cut in the corresponding instance granularity tree and thus to the same instance granularity level (see also [19]).

This example clearly demonstrates the problems with cumulative-constitutively organized material entities: different particular instances of the same type of material entity (e.g. the type *MOLECULE*) can belong to different cuts and thus different levels of instance granularity. In other words, the extensions of some types of material entities *transcend* the boundary between different levels of instance granularity (Fig. 7B,C right). Therefore, and contrary to constitutively organized material entities, for a given cumulative-constitutively organized entity the instance granularity tree and the type granularity tree *cannot* share the same topology. Transforming the *instance granularity tree* of a cumulative-constitutively organized material entity into its corresponding *type granularity tree* is thus not as straight forward as it is for constitutively organized material entities [19].

The applicability of the single inheritance principle is also affected: not only its general global application is problematic, as we already discussed further above with respect to the implications of a constitutive granularity, but for cumulative-constitutively organized material entities its application relative to a given level of type granularity is also problematic and not directly practicable, because the extensions of categories of cumulative-constitutively organized material entities *transcend* the boundary between different instance granularity levels.

Introducing some sort of container category ‘anatomical group’ that allows the representation of anatomical structures that are composed of direct proper parts belonging to different levels of granularity (see e.g. [37]) does not solve this problem either. It merely demarcates the problematic cumulative-constitutively organized anatomical structures from the unproblematic constitutively organized ones.

One intuitive solution for transforming the instance granularity tree into a type granularity tree for a cumulative constitutive granularity is a *sortation-by-type* ([19]) that can best be understood as a granular sedimentation of all the instances of one type to the finest level of granularity that one of them occupies, resulting in the types being ranked according to the finest level of granularity at which their instances occur (Fig. 7C right). Sortation-by-type alone, however, is insufficient, since it has undesirable side effects regarding the consistency of granular partitions of types of material entities ([19]). If the assumption that all material entities of a given level of granularity must sum to the complete whole should also apply to cumulative-constitutively organized entities, we required categories for the trans-granular instantiations that the extended BFO does not provide: we required a category for molecules at levels coarser than the molecular level and a category for cells at levels coarser than the cellular level of type granularity (marked ‘???’ in Fig. 7C right).

For instance the ECM of a multicellular organism belongs, through sortation-by-type and in virtue of its respective instance granularity tree, not only to the organ type granularity level (alongside with the organism's organs), but also to the cellular type granularity level alongside with the organism's cells and to the molecular type granularity level alongside with the organism's other molecules (Fig. 7B,C right). Only this way, the abovementioned assumption can be maintained. Referring to ECM as ‘fiat organ part’ or ‘fiat multicellular organism part’ within the cellular type granularity level does not adequately account for the *trans-granular multiple instantiation* that results from a cumulative constitutive granularity, neither does restricting a type to a specific level of a type granularity. Therefore, another rule is required for the transformation of an instance to a type granularity tree. This involves the notion of *granular representation*, which should allow maintaining the single inheritance principle across different levels of granularity.

#### Granular Representation and the Single Inheritance Principle

Due to the *trans-granular* multiple instantiation that is characteristic for cumulative-constitutively organized material entities, for a given type of entity different categories are required at different levels of granularity. These categories cannot be derived from extended BFO's categories of material entity. Thus, if ontologies shall cover also cumulative-constitutively organized material entities, they must somehow integrate different levels of type granularity and cannot be restricted to a single type granularity level. What does that imply for ontology design and the organization of top-level categories of material entity in a formal top-level ontology like BFO? Must the single inheritance principle necessarily be abandoned for ontologies covering also cumulative-constitutively organized material entities?

Ontologies that integrate several type granularity levels are faced with the following situation: not only does every cell of a multicellular organism instantiate ‘molecule aggregate*^cell^*’, which is the class of those molecule aggregates that are cells and thus a specific subcategory of ‘molecule aggregate’, but every instance of this ‘molecule aggregate*^cell^*’ instantiates also the category ‘cell’. In other words, *some* molecule aggregates, which we here call ‘molecule aggregate*^cell^*’, are cells and all cells are molecule aggregates. Therefore, these two categories, ‘cell’ and ‘molecule aggregate*^cell^*’, share the same extension (i.e. every entity that instantiates ‘cell’ also instantiates ‘molecule aggregate*^cell^*’, and vice versa) and thus refer to the same type of material entity. If according to the single inheritance principle two different categories are instantiated by the same particular material entity, one category must be subsumed under the other. In the example above, however, the two categories share the same extension, so how can one of these two categories be subsumed under the other? They would have to subsume each other, thereby implying that they are *ontologically* identical – two mappings of the same real world entity ([19]).

However, in this context it is important to keep in mind that the instantiation relation between a particular cell and the ‘object’ subcategory ‘cell’ entails the reference to the cellular level of granularity as an implicit condition: this entity is an object *only* at the cellular level of granularity. At all other levels of granularity it is not an object. The other instantiation relations of that particular cell entail references to other granularity levels as their implicit conditions (*A1*): For all entities *X* of type *CELL* holds that *X* instance_of ‘object aggregate’ iff granularity level<cellular; for all entities *X* of type *CELL* holds that *X* instance_of ‘object’ iff granularity level = cellular; for all entities *X* of type *CELL* holds that *X* instance_of ‘fiat object part’ iff granularity level>cellular.

As a consequence, which of the many granular representations one uses for referring to a particular cell directly depends on the level of granular focus that one has chosen. Whereas this multiple instantiation does not violate the single inheritance principle, its implications for the corresponding class-subclass relations does: when expressing a possible class-subclass relation between the category ‘cell’ and BFO's category ‘object’, one must also entail the reference to the cellular level of granularity as an implicit condition of this relation, as does a possible class-subclass relation to BFO's ‘object aggregate’ implicitly entail the reference to all granularity levels finer than the cellular level, and a relation to BFO's ‘fiat object part’ the reference to all granularity levels coarser than the cellular level (*A2*): ‘cell’ is_a ‘object aggregate’ iff granularity level<cellular; ‘cell’ is_a ‘object’ iff granularity level = cellular; ‘cell’ is_a ‘fiat object part’ iff granularity level>cellular. This, however, would imply that BFO's top-level categories of material entity are not mutually disjoint.

The specific categories ‘atom aggregate*^cell^*’, ‘molecule aggregate*^cell^*’, ‘cell’, and ‘fiat organ part*^cell^*’, which share the same extension, can be subsumed under BFO's top-level categories of material entity requiring corresponding conditional extensions (A3): ‘atom aggregate*^cell^*’ is_a ‘object aggregate’ iff granularity level = atomic; ‘molecule aggregate*^cell^*’ is_a ‘object aggregate’ iff granularity level = molecular; ‘cell’ is_a ‘object’ iff granularity level = cellular; ‘fiat organ part*^cell^*’ is_a ‘fiat object part’ iff granularity level = organ level.

How can we organize these relations within a single ontology while still following the single inheritance principle? In case of constitutively organized material entities this problem is sidestepped by restricting each ontology to cover only entities that belong to the same level of granularity and using cross-ontology relations to refer to entities from different granularity levels. Due to the trans-granular multiple instantiation of cumulative-constitutively organized material entities discussed above, this elegant solution cannot be applied to a cumulative constitutive granularity.

Whereas the classes ‘atom aggregate*^cell^*’, ‘molecule aggregate*^cell^*’, ‘cell’, and ‘fiat organ part*^cell^*’ share the same extension and thus are representations of the same general type CELL (see also Fig. 2), due to the implicit granular condition of reference, epistemologically they nevertheless cannot be strictly synonymized. Therefore, instead of relating these categories with one another using the subsumption relation one must apply the specific relation of granular representation (i.e. has_coarser_representation, has_finer_representation; see also Fig. 2). Using this relation, one can treat ‘atom aggregate*^cell^*’, ‘molecule aggregate*^cell^*’, ‘cell’, and ‘fiat organ part*^cell^*’ as different representations of the same type CELL within different granular levels – they are the granular representations of a cell at the atom, molecule, cell and organ level of granularity. Therefore, the conditional extension belongs to the granular representation relation and not to the class-subclass relation. As a consequence, we can conclude that the second set of assertions (A2) is incorrect and they must be modified to: CELL has_granular_representation ‘atom aggregate*^cell^*’ iff granularity level = atomic; CELL has_granular_representation ‘molecule aggregatecell’ iff granularity level = molecular; CELL has_granular_representation ‘cell’ iff granularity level = cellular; CELL has_granular_representation ‘fiat organ part*^cell^*’ iff granularity level = organ.

In this way, the different categories relate to one another in dependence of a given granularity scheme and at the same time the single inheritance policy can be applied across multiple granularity levels. In case one is exclusively dealing with constitutively organized material entities, *granular representation* serves as the cross-ontology relation between the different categories belonging to different levels of granularity that are instantiated by the same particular material entity. One can switch between the different granular *representations* of a given type of material entity and can distinguish them although they refer to the same type of entity. Which representation is used for referring to a real world entity results from the combination of the entity type and the scale or level of granularity at which it is considered ([19]; see *sgrG* granularity type, [27], [38]).

#### Cross-Granular Instantiation and Granular Representation

With the notion of *granular representation* we can now deal with the problem of trans-granular instantiations of the same type of material entity in a cumulative constitutive granularity. If one uses different granular representations for referring to the instances of the same type of material entity that belong to different levels of the instance granularity tree, a specific type of material entity could belong to more than one type granularity level and the instance granularity tree could be transformed to the type granularity tree (see Fig. 7C). As a matter of fact, contrary to the instance granularity tree, the corresponding type granularity tree would then not be restricted to object entities, but will include other top-level categories as well (compare Fig. 7C right, the entities marked ‘???’, and right). *Granular representations* are thus required in order to account for the trans-granular instantiations of cumulative-constitutively organized types of material entities.

### Complementing the Extended Basic Formal Ontology to also Cover Cumulative-Constitutively Organized Material Entities

In order to be able to transform instance granularity trees of cumulative-constitutively organized material entities into type granularity trees we must provide additional categories to accommodate for trans-granular multiple instantiation. These additional categories must be used in combination with the notion of granular representation. We require for instance a category for representing extra-cellular molecules at levels coarser than the molecular level and a category for representing cells occurring outside from organs at levels coarser than the cellular level of type granularity (see Fig. 7C right, categories marked ‘???’). What are these additional categories?

**Figure 8:**
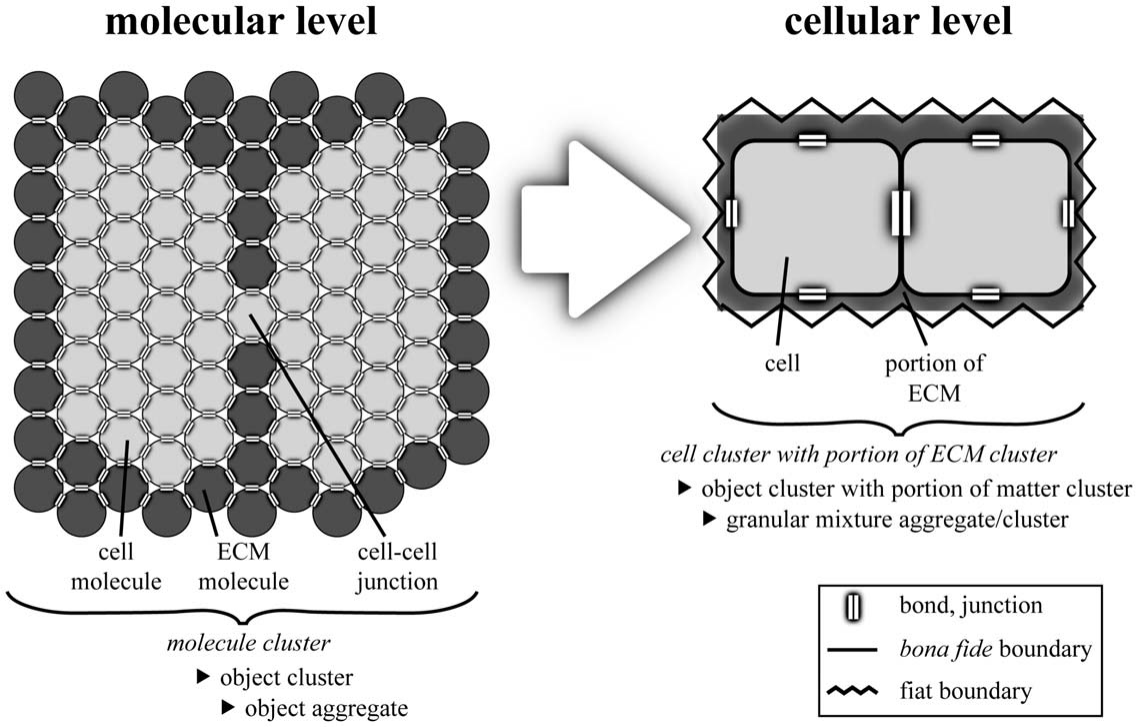
A Cluster of Cells at the Molecular and the Cellular Level of Granularity. Left - A material entity that is a cluster of molecules at the molecular level of granularity. The molecule cluster constitutes two cells embedded in extracellular matrix (ECM). The two cells are connected with each other via a cell-cell junction. The molecules form a molecule cluster since they are connected with each other via molecular bonds. Right - The same material entity at the cellular level of granularity in which it is a cell cluster with portion of ECM cluster. The cell molecules constitute two cells that are connected via a cell-cell junction, thus forming a cell cluster. The ECM molecules form a portion of ECM that is a fiat entity.

In a constitutive granularity, at the cellular level a particular cluster of molecules constitutes either a ‘fiat cell part’, ‘fiat cell part cluster’, ‘cell’, ‘cell with fiat cell part cluster’, or a ‘cell cluster’. This is no surprise since every molecule is part of some object of the next coarser level of constitutive granularity. In a cumulative constitutive granularity, however, this is not the case. Here, molecule clusters exist that neither instantiate ‘fiat cell part’, nor one of the other categories of the cellular level mentioned above. This is due to the fact that not every molecule is necessarily part of some cell (e.g. ECM, blood plasma, lymph; Fig. 8). But what do these molecule clusters constitute instead at coarser levels of granularity? What is ECM at the cellular level?

At the cellular level of granularity, ECM is beyond the coverage of the top-level categories discusses so far. One would have to switch the level of granular focus to coarser levels of granularity in order to find an adequate category for ECM, like for instance ‘fiat organ part’ or ‘fiat multicellular organism part’.

Some molecule clusters constitute cell clusters *and* the ECM in which the cells are embedded. Consequently, these would have to be referred to as ‘cell with fiat organ part cluster’ at the cellular level of granularity (Fig. 8). In multicellular animals most cell clusters are actually such cell with fiat organ part clusters. A category ‘cell with fiat organ part cluster’, however, as the reference to fiat organ parts implies, requires the reference to a level of granularity that is coarser than the cellular level, irrespective of the fact that the actual level of granular focus is on the cellular level. This is rather awkward and in some cases also not desirable. For instance, if one is dealing with a tissue sample of unknown origin and one has no information regarding the organ from which it has been taken, or if one wants to describe a tissue sample without reference to the organ level at all. Moreover, if the assumption that all parts that share the same instance granularity level exhaustively sum to the whole should also apply to cumulative-constitutively organized material entities, we required a category for referring to ECM at the cellular level, because ECM does exist at that level and it is not part of any cell.

#### Portion of Matter

In order to be able to refer to ECM at levels of granularity coarser than the molecular level we introduce the category ‘portion of matter’ (c. f. *amount of matter* sensu [39], *masses* sensu [40], *portion of unstructured/structured stuff* sensu [41], and to a limited degree also to *body substance* sensu [42] and *portion of body substance* sensu [43]).

#### Definition for ‘portion of matter’

*A material entity that is not demarcated by any physical discontinuities. At some finer level of granularity it is an object aggregate, at some coarser level of granularity it is a fiat object part, but at this level of granularity it is neither.*

#### Explanation

What is a molecule cluster at the cellular level? Contrary to the molecular level, at the cellular level of granularity the boundary of each molecule is outside of the level of granular focus. What forms a discontinuous cluster of countable individual molecules and thus *bona fide* objects at the molecular level, is a continuous and non-countable *molecular substance* at coarser levels of granularity. At these coarser levels, the individual molecules cannot be differentiated and demarcated anymore and the cluster as a whole possesses only fiat inner boundaries.

This granularity dependence of inner boundaries can be characterized with the distinction of *topological adherence and topological coherence* (see Table 3; [4]): topological adherence between the objects of an object cluster of a given level of granularity is treated as topological coherence at coarser levels. Therefore, objects within the cluster cannot be differentiated anymore at coarser levels of granularity, because their *bona fide* boundaries disintegrate at these coarser levels and only fiat boundaries remain. As a consequence, at levels coarser than the molecular level of granularity a molecule cluster is not necessarily an object cluster anymore (see above, Fig. 8). Instead, at these levels a molecule cluster often constitutes a fiat entity. While this does not pose any problem for constitutively organized material entities, since any molecule cluster is part of an object of the adjacently coarser level of granularity and thus can be referred to as a fiat object part at this level, it does for cumulative-constitutively organized material entities.

The here proposed distinction of countable objects and non-countable *portions of matter* resembles the linguistic distinction of the grammatical concepts of *count-nouns* and *mass-nouns* [18]. Count-nouns, like for instance table, stone or cell, can be used in the singular and in the plural, whereas mass-nouns, like for instance gold, blood or tissue, are typically considered to be invariable in grammatical number [44], [45]. If mass-nouns are treated as non-singular terms (see [46]), however, the distinction of count-nouns and mass-nouns correlates also semantically with the here proposed distinction of countable objects and non-countable *portions of matter*. As mass-nouns, and because portions of matter are fiat entities, they require some arbitrary but standardized quantification measure (e.g. *a glass* of water, *1m^3^* of soil, *25g sugar*, *250ml orange juice, 1km^2^ forest*).

#### Examples

Taking a cluster of H_2_O molecules as an example: while at the molecular level of granularity the individual H_2_O molecules of a particular H_2_O cluster are distinguishable and countable. Their representation (not their ontological nature!) changes at coarser levels of granularity. Here, the individual molecules of the H_2_O cluster are not countable anymore (i.e. they are outside of the level of granular focus) and the cluster is represented as a *portion of water*, which is a specific type of *portion of molecular substance*, which at its turn is a subcategory of *portion of matter*. Thus, it is the *granular representation* of the real world entity type *H_2_O CLUSTER* that is different at different levels of granularity [19], [27].

In a similar manner one can differentiate between a particular cytosol molecule cluster at the molecular level and a *portion of cytosol* at the organelle level of granularity (Table 4). Both are granular representations of the same type of material entity (i.e. *CYTOSOL*). We use the former when we are interested in the actual molecular composition of the cytosol and the latter when we lack knowledge or simply do not care about the exact number, topological positions, and types of molecules that constitute it. In general, we can say that at granularity levels coarser than the molecule level, all fluids and extracellular substances of an organism, as for instance dentin, are *portions of molecular substance*.

Some types of portions of molecular substance are not part of any organelle (e.g. blood plasma, cerebrospinal fluid, cytosol, gastric juice, lymph, mucus, peritoneal fluid, saliva, seminal plasma, secret in a multicellular gland, urine) and thus cannot constitute a fiat organelle part. At the cellular level, ECM is not a fiat cell part either (Table 4). Since they lack *bona fide* boundaries, these various types of portions of matter nevertheless constitute fiat entities. But these fiat entities lack their parthood counterpart in the respective granularity levels, since they are fiat parts of objects at a coarser level of granularity and therefore cannot be referred to as fiat object parts at these levels of granular focus.

**Table 4:**
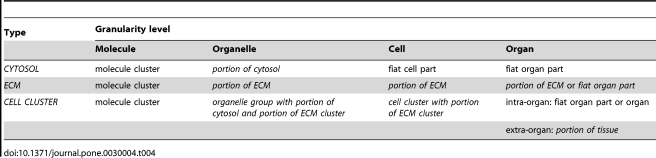
Three Types of Material Entity and their different Granularity Representations at Four Levels of Granularity.

On coarser granularity levels object aggregates do not possess their own boundaries and referring to them using the ‘fiat object part’ category requires the reference to objects at coarser levels. In this respect, portions of matter are comparable to negative entities (i.e. immaterial entities, such as tunnels, caves, and hollows) that also do not possess their own boundaries. This, however, is not problematic, since the expression *portion of matter* as proposed here already implies fiatness of the respective entity without requiring reference to coarser objects. Consequently, though at the cellular level ECM does not constitute a fiat cell part, as a *portion of ECM* it is nevertheless designated as a fiat entity (Fig. 8). In this way, one can refer to ECM within a cellular level of granular focus without having to refer to any categories from coarser levels of granularity. One could also say that, ontologically, a portion of matter is situated in the border region between an object aggregate at a finer level of granularity and a fiat object part at a coarser level. A portion of matter is in a no man's land, taking in a granular intermediate state in which it is neither an object aggregate nor a fiat object part, but something in between.

Since portions of matter are representations of object aggregates at levels of granularity coarser than their respective object level, object aggregates involving different types of objects are not ontologically distinguished at the level of ‘portion of matter’ from those involving only one type of object. As a consequence, at this taxonomic level solutions are not distinguished from pure stuffs: a portion of lemonade and a portion of pure distilled water are both portions of matter, irrespective of their different molecular composition.

#### Additional Top-Level Categories resulting from Portion of Matter as another Building Block

In a constitutive granularity, every material entity either is an object, a fiat object part, or any possible combination and topological distribution of aggregates thereof. Therefore, ‘object’ and ‘fiat object part’ represent the basic building blocks of the top-level categories of constitutively organized material entities [4]. The top-level categories of material entity, at their turn, can be distinguished by the composition and topological distribution of these building blocks. The set of possible constellations of the two building blocks exhaustively covers all possible types of constitutively organized material entities [4].

**Figure 9:**
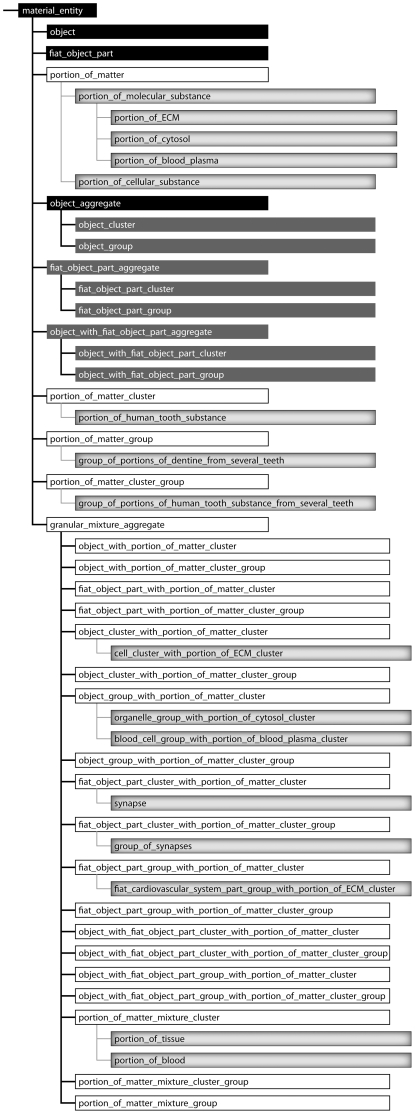
Taxonomy of Top-Level Categories of Constitutively and Cumulative-Constitutively Organized Material Entities. A taxonomy of top-level categories of material entity and important subcategories that can be distinguished in constitutively and cumulative-constitutively organized material entities. *Black boxes indicate Basic Formal Ontology (BFO) categories of material entity, dark grey boxes the additional categories suggested by* [4], *white boxes additional categories required for accommodating all cumulative-constitutively organized material entities, and shaded grey boxes examples for specific subcategories involving portions of matter as building blocks.*

Unfortunately, the set of possible constellations of the two building blocks does not exhaustively cover all possible types of cumulative-constitutively organized material entities. The examples from above demonstrate that in a cumulative constitutive granularity we require ‘portion of matter’ as an additional building block for all those cases in which objects are not part of objects of the adjacently coarser level of granularity. Adding ‘portion of matter’ as another building block yields a variety of additional top-level categories of material entity that result from all possible constellations of ‘portion of matter’ with itself and with the other two building blocks and from their possible topological distributions (Fig. 9).

One can distinguish two basic types of additional categories: (i) material entities that involve portions of matter but are composed of objects of the same granularity level, and (ii) material entities that involve portions of matter that are composed of objects or parts of objects of more than one granularity level and thus are granular mixture aggregates (for definitions see Table S1). The former comprises ‘portion of matter group’, which is a group of portions of matter that are spatially separated (e.g. *group of portions of dentine* of an individual human being, with each portion belonging to a separate tooth), ‘portion of matter cluster’, which is a cluster of spatially distinct portions of matter entities (e.g. *portion of human tooth substance cluster*, which consists of a cluster of spatially distinct portions of matter, i.e. enamel, dentin, cementum), and ‘portion of matter cluster group’, which is a group of spatially separated portion of matter cluster entities (e.g. group of all *portions of human tooth substance clusters* of an individual human being). All other additional categories are granular mixture aggregates (definitions see Table S1).

#### Definition for ‘granular mixture aggregate’

*A material entity that is a mereological sum of several separate material entities of different granularity levels and possesses non-connected boundaries.*

#### Explanation and Examples

Characteristic for the cumulative constitutive granularity is the existence of various types of material entities that are composed of objects of different levels of granularity, like object clusters that are embedded in a portion of matter, as for instance cells in extracellular matrix (i.e. ECM). The distinction of count-nouns and mass-nouns mentioned above also applies to ECM: At granularity levels coarser than the molecule level we refer to ECM using the mass-noun *portion of ECM*. As a consequence, at the cellular level of granularity a cluster of cells embedded in ECM is a *cell cluster with portion of ECM cluster*, which is a subcategory of ‘object cluster with portion of matter cluster’ (definition see Table S1). When considering only single cells and their surrounding ECM, they are *cell with portion of ECM cluster* entities, which belong to the subcategory ‘object with portion of matter cluster’, and spatially separated groups of such clusters are *cell with portion of ECM cluster group* entities, which belong to the category ‘object with portion of matter cluster group’.

In case one differentiates an organelle level from a molecule and a cell level of granularity, one also has to deal with molecules that are not part of any organelle (e.g. ECM, cytosol). In this case, a cell is an *organelle group with portion of cytosol cluster* at the organelle level of granularity, which is a subcategory of ‘object group with portion of matter cluster’ (definition see Table S1). Human blood at the cellular level is another example for an ‘object group with portion of matter cluster’. Blood at the cellular level is a *blood cell group with portion of blood plasma cluster*, since the individual blood cells are spatially separated from one another and surrounded by blood plasma, which, at its turn, is a molecule cluster at the molecular level of granularity.

Sometimes, it is necessary to only refer to specific parts of a cumulative-constitutively organized material entity. For instance, when talking about the group of blood vessels of a human lung or of other human organs. Blood vessels in humans are always accompanied by ECM and form a continuously connected network that constitutes the cardiovascular system, which is a *bona fide* object. The blood vessels of the lung therefore represent an aggregate of specific parts of this system, each of which are demarcated from the remaining cardiovascular system by fiat boundaries. As the blood vessels of the lung are spatially separated distinct fiat parts of the system as a whole, which, as fiat parts, are not all directly connected with each other (only indirectly via the remaining vessels of the cardiovascular system), they form a *fiat object part group* that is accompanied by some *portion of ECM*. The same applies to the vessels of other human organs. As such, they are *fiat cardiovascular system part group with portion of ECM clusters*, which is a subcategory of ‘fiat object part group with portion of matter cluster’ (definition see Table S1). When considering their spatial distribution and topological constellation within a human individual, they form a group of *fiat cardiovascular system part group with portion of ECM clusters*, which is a subcategory of ‘fiat object part group with portion of matter cluster group’ (definition see Table S1).

A synapse is an example of a *fiat object part cluster with portion of matter cluster* entity (definition see Table S1). A synapse is commonly considered to be an intercellular junction composed of the presynaptic zone of a neuron and the postsynaptic zone of another neuron, muscle cell or secretory cell, with an intervening synaptic cleft between these two zones. The two pre- and postsynaptic zones of a synapse are fiat cell parts that form a *fiat cell part cluster*, whereas the cleft is filled with some *portion of intercellular fluid*. Thus, a synapse is a *fiat object part cluster with portion of matter cluster* entity. Other intercellular junctions, as for instance desmosomes, are also examples of *fiat object part cluster with portion of matter cluster* entities, since they are physical junctions in which transmembrane proteins from two adjacent epithelial cells bridge the space between them, forming a *fiat object part cluster*, and the space between them is filled with some *portion of extracellular liquid*. The topological constellation of several synapses or several desmosomes of an individual human being forms a group of *fiat object part cluster with portion of matter clusters*, which is a subcategory of ‘fiat object part cluster with portion of matter cluster group’ (definition see Table S1).

In order to identify all major categories of cumulative-constitutively organized material entities, the building block ‘portion of matter’ also has to be combined with itself, involving portions of matter from objects belonging to different granularity levels. This results in portion of matter mixture aggregates that are granular mixtures of object aggregates at levels of granularity coarser than the level in which their constituting object entities can be demarcated. For instance cell clusters embedded in ECM at granularity levels coarser than the cellular level (Table 4). Because the cells of a cell cluster are objects at the cellular level, they constitute a *portion of cellular substance* at levels of granularity coarser than the cellular level – at these coarser levels the individual cells cannot be differentiated anymore [19]. Thus, at levels coarser than the cellular level a cluster of cells embedded in ECM is a portion of cellular substance that is clustered with a portion of ECM, constituting a *portion of tissue* (c.f. [42]). As a consequence, a portion of tissue is an aggregate mixture of two different types of portion of matter entities, with the portion of matter entities forming object aggregates at different levels of granularity.

A *portion of tissue* therefore is a subcategory of ‘portion of matter mixture cluster’ (definition see Table S1). As a consequence, tissue belongs to a different category than body liquids. They are ontologically clearly distinct types of material entities (c.f. [43]). The same holds for blood, which is composed of blood cells and blood plasma. At the cellular level of granularity, a portion of blood is a *blood cell group with a portion of blood plasma cluster*, but at granularity levels coarser than the cellular level the blood cell group is a *portion of cellular substance*. A *portion of blood*, thus, is a cluster of two different portions of matter (i.e. *portion of blood cell substance* and *portion of blood plasma*). Lymph is another example for an aggregate of two different portions of matter (i.e. *portion of lymphocytes substance* and *portion of lymph plasma*). Since all these aggregates are granular mixtures, tissue, blood and lymph belong to the same top-level category of material entity (contradicting [43]), but are clearly distinguished from body substances, such as blood plasma, cytosol or ECM, that exclusively consist of material entities of the same granularity level (c.f. *structured* and *unstructured stuff* sensu [41]).

## Discussion

### An Integrated Taxonomy of Top-Level Categories of Material Entity

The set of top-level categories of material entity suggested above (see also additional definitions in Table S1) is more comprehensive than the current set of BFO's top-level categories and meets the requirement of exhaustively covering all possible types of cumulative-constitutively organized material entities. However, the additional categories and the way they are organized make the resulting top-level taxonomy complex (Fig. 9). Part of this increased complexity reflects the complex reality we are dealing with, part is owed to the particular taxonomic hierarchy employed in this specific taxonomy.

When dealing with sufficiently complex real entities, there is always more than one way of organizing their respective types within an ontologically sound taxonomy of top-level categories. When developing an ontology one is thus often faced with the choice between two or more alternative ways of organizing the respective categories within a taxonomic hierarchy that are equally consistent and ontologically correct. At the end of the day, the preference of one taxonomy of top-level categories over all other possible alternatives to be implemented in a particular ontology is a matter of design and thus rests on pragmatic rather than purely ontological considerations. Therefore, it is legitimate to ask whether it is possible to organize the top-level categories of material entity in a simpler and clearer way that is easier to manage and navigate and more suitable for ontology applications.

When discussing the pros and cons of alternative hierarchies, one must keep in mind the specific function of a formal top-level ontology. It must provide a top-level taxonomy for many different domain reference ontologies and application ontologies. Different domains have different demands on top-level categories, and so do different application ontologies. Some of the here proposed additional categories, as for instance ‘object with fiat object part group with portion of matter cluster’ or ‘portion of matter mixture group’, might not be relevant to science at all, because no real correlate exists that is important to science, but have been included for matters of completeness, whereas other categories, as for instance ‘object with fiat object part cluster with portion of matter cluster’ or ‘fiat object part cluster with portion of matter cluster group’ will only be relevant to some ontologies. Other categories, however, as for instance ‘object’, ‘fiat object part’ or ‘portion of matter’, are relevant to almost all ontologies that are dealing with biological material entities. Therefore, it is desirable to have a *small* number of top-level categories that are direct sub-classes of ‘material entity’ and that exhaustively cover all types of material entity. These foundational categories of material entity provide a very general differentiation of basic types of material entity. However, since most domain reference ontologies require a more specific differentiation, a good foundational top-level ontology provides a set of additional top-level categories that can be used as needed by domain and application ontologies – again, with a preferably small number of taxonomically more basic categories.

BFO currently (i.e. BFO Version 1.1) differentiates three foundational categories of material entity: (i) ‘object’, (ii) ‘fiat object part’, and (iii) ’object aggregate’ and does not specify any additional categories. In [4] we have shown that this basic scheme is not exhaustive and needs to be extended by two further foundational categories (i.e. ‘fiat object part aggregate’ and ‘object with fiat object part aggregate’). Moreover, we have argued that BFO's top-level categories of material entity are also not differentiated enough to meet the requirements of many domain reference ontologies, and therefore we suggested six additional categories (i.e. differentiation of all aggregates of material entities into *clusters* and *groups*; [4]). Above we argue that in order to accommodate cumulative-constitutively organized material entities, further foundational and additional categories must be included. All the categories we suggested in [4] and in the present paper represent different top-level categories of material entity that are not yet covered or differentiated by BFO.

However, the resulting complex taxonomy of ten foundational and 25 additional categories of material entity can be significantly simplified by introducing a new foundational category ‘material entity aggregate’ with the direct subcategories ‘material entity cluster’ and ‘material entity group’ (definition see Table S1), under which all categories of material entity aggregates can be subsumed – including BFO's ‘object aggregate’.

#### Definition for ‘material entity aggregate’

*A material entity that is a mereological sum of separate material entities and possesses non-connected boundaries.*

#### Definition for ‘material entity cluster’

*A material entity aggregate that is a mereological sum of separate material entities, which adhere to one another through chemical bonds or physical junctions that go beyond gravity.*

#### Definition for ‘material entity group’

*A material entity aggregate that is a mereological sum of scattered (i.e. spatially separated) material entities, which do not adhere to one another through chemical bonds or physical junctions but, instead, relate to one another merely on grounds of metric proximity. The material entities are separated from one another through space or through other material entities that do not belong to the group.*

#### Explanation

**Figure 10:**
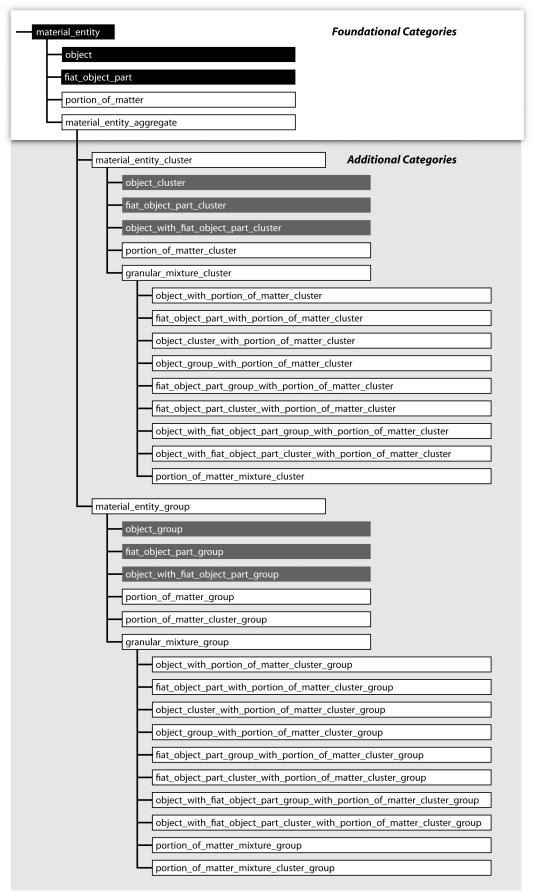
Integrated and Simplified Taxonomy of Top-Level Categories of Material Entity. A taxonomy of top-level categories of material entity and important subcategories that can be distinguished in constitutively and cumulative-constitutively organized material entities. By introducing the category ‘material entity aggregate’ one can reduce the number of *foundational categories* that exhaustively cover all possible types of constitutively and cumulative-constitutively organized material entities to four. All other categories represent *additional categories* that are organized into three hierarchical levels and subsumed under the foundational category ‘material entity aggregate’. All clusters of material entities are subsumed under ‘material entity cluster’ and all groups under ‘material entity group’ respectively. *Black boxes indicate Basic Formal Ontology (BFO) categories of material entity, dark grey boxes the additional categories suggested by* [4], *and white boxes additional categories required for accommodating all cumulative-constitutively organized material entities.*

The introduction of these three additional categories reduces the number of foundational categories to four: (i) ‘object’, (ii) ‘fiat object part’, (iii) ‘portion of matter’, and (iv) ‘material entity aggregate’ (Fig. 10). As a consequence, all other top-level categories would be subsumed under the two direct subcategories of ‘material entity aggregate’. With the introduction of ‘granular mixture cluster’ and ‘granular mixture group’, the resulting taxonomy of material entity categories would be organized into four levels. The top-level comprises the abovementioned four foundational categories, one of which entails all aggregate entity categories. This latter foundational category includes two direct subcategories, which differentiate between clusters and spatially separated groups of material entity aggregates. The third level comprises five top-level categories of material entity aggregates in case of clusters and six in case of groups, including ‘granular mixture cluster’ and ‘granular mixture group’. As a consequence, all of the granular mixture aggregate subcategories discussed above belong to the fourth level of the hierarchy (Fig. 10; definitions see Table S1).

Compared to BFO's current top-level categories of material entity, this simplified taxonomy has four instead of three top-level categories, two of which overlap with BFO (i.e. ‘object’, ‘fiat object part’). Contrary to BFO, however, it manages to accommodate for all types of constitutively as well as all types of cumulative-constitutively organized material entities. Moreover, with the suggestion of second and third level top-level categories, the simplified taxonomy provides three levels of differentiation that can be used by domain reference ontologies and application oriented ontologies as they may need as top-level templates.

### Conclusions

**Figure 11:**
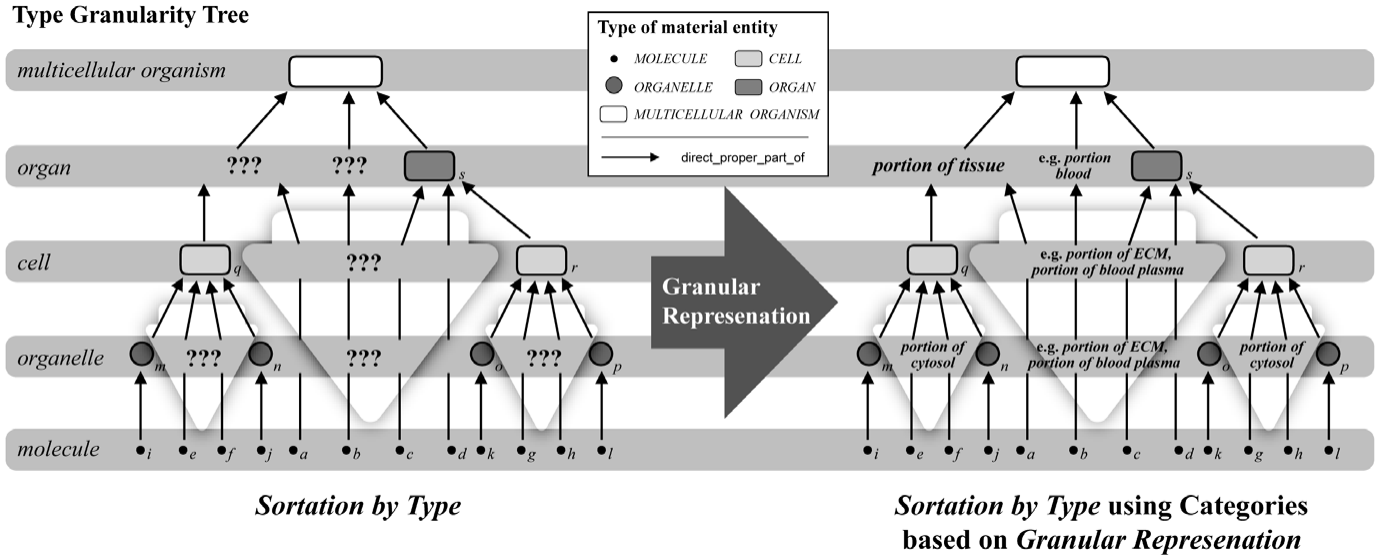
Sortation by Type using Categories based on Granular Representation. Left: Following the rule of sortation-by-type and applying a more complicated granularity scheme (Vogt 2010) one infers this type granularity tree from the instance granularity tree shown in Fig. 7. This results in types of entities belonging to type granularity levels for which BFO provides no respective categories as templates for granularity specific ontologies (here marked ‘???’): BFO (version 1.1) provides no category for molecules that are not part of any cell, since they are neither a cell (i.e. BFO's ‘object’), nor a cell aggregate (i.e. BFO's ‘object aggregate’) or fiat cell part (i.e. BFO's ‘fiat object part’). Right: By applying the notion of *granular representation* and the here proposed categories of *portion of matter* and *portion of matter aggregate*, the transformation of the instance granularity tree into a type granularity tree can be completed. With these additional categories and the notion of granular representation one can account for the effects of trans-granular multiple instantiation in cumulative-constitutively organized material entities.

Further above we have argued that the notion of granular representation is required in order to account for the trans-granular multiple instantiations of cumulative-constitutively organized material entities. As a consequence, we suggested additional categories for BFO. Now we can complete the transformation of instance granularity trees to type granularity trees for cumulative-constitutively organized material entities (Fig. 11) by specifying the categories for extra-cellular molecules at levels coarser than the molecular level and the categories for cells occurring outside from organs at levels coarser than the cellular level of type granularity. Obviously, and contrary to constitutively organized material entities, the type granularity tree that results from the compositional partitioning of a cumulative-constitutively organized material entity is not restricted to object entities, but also includes portions of matter and portion of matter aggregates (Fig. 7C left, Fig. 11 right). The here proposed notion of granular representation thus allows to maintain the clear distinction of fixed levels of a compositional granularity [19] also for a type granularity tree of cumulative-constitutively organized material entities (contrary to [39], who have explicitly rejected any notion of a fixed set of levels of granularity) and still accommodate for the specificities of biological material entities that result from their cumulative constitutive granularity.

It has been acknowledge before that types of material entity could be recognized “[…] *that are not explicitly identified in the BFO taxonomies or definitions that have been given* […]” ([11], p. 70). However, these types of material entity would “[…] *lack salience and are systematically irrelevant for a principled analysis of the ontology of scientific domains* […]” and are therefore “[…] *intentionally not included in any existing definition sets, taxonomies or implementations of BFO*” ([11], p. 71). Above we have shown that the current top-level categories of material entity of BFO do not only fail to exhaustively cover all types of scientifically relevant types of cumulative-constitutively organized material entities, but also that they do not allow for developing domain ontologies that are consistent with the single inheritance policy if these ontologies are supposed to cover also scientifically relevant types of cumulative-constitutively organized material entities. By extending BFO with the here proposed additional top-level categories of material entity and those categories we proposed elsewhere [4], BFO will cover *all* types of material entity, while still maintaining the single inheritance principle.

Moreover, with the here proposed notion of granular representation and the additional categories of material entity that involve portions of matter, formerly problematic types of material entities are covered as well. We provide a clear and ontologically consistent classification for body liquids (e.g. cytosol, blood plasma), body substances (e.g. ECM, dentin) and granular mixture portions (e.g. blood, tissue, lymph), which are otherwise usually treated as special cases that could not be subsumed under the current categories of BFO (Version 1.1).

## Supporting Information

Table S1 Definitions of additional Top-Level Categories of Material Entity required for Cumulative-Constitutively organized Material Entities.
(DOC)

## References

[1] Stevens R, Goble CA, Bechhofer S (2000). Ontology-based knowledge representation for bioinformatics.. Briefings in Bioinformatics.

[2] Bard J (2003). Ontologies: Formalising biological knowledge for bioinformatics.. BioEssays news and reviews in molecular cellular and developmental biology.

[3] Bard JBL, Rhee SY (2004). Ontologies in biology: design, applications and future challenges.. Nature Reviews Genetics.

[4] Vogt L, Grobe P, Quast B, Bartolomaeus T (2011). Top-Level Categories of Constitutively Organized Material Entities - Suggestions for a Formal Top-Level Ontology.. PLoS ONE.

[5] Smith B, Munn K, Papakin I (2004). Bodily systems and the spatial-functional structure of the human body.. Studies In Health Technology And Informatics.

[6] Rosse C, Kumar A, Jose LV, Mejino J, Cook DL (2005). A Strategy for Improving and Integrating Biomedical Ontologies.. American Medical Informatics Association Symposium.

[7] Brinkley JF, Suciu D, Detwiler LT, Gennari JH, Rosse C (2006). A framework for using reference ontologies as a foundation for the semantic web.. In: AMIA 2006 Annual Symposium proceedings.

[8] Smith B, Kusnierczyk W, Schober D, Ceusters W (2006). Towards a Reference Terminology for Ontology Research and Development in the Biomedical Domain.. In:Proceedings of KR-MED'2006, Studies in Health Technology and Informatics, Vol.

[9] Schulz S, Boeker M, Stenzhorn H, Niggemann J (2009). Granularity issues in the alignment of upper ontologies.. Methods of Information in Medicine.

[10] Smith B, Kumar A, Bittner T (2005). Basic Formal Ontology for Bioinformatics.. Journal of Information Systems.

[11] Spear AD (2006). Ontology for the Twenty First Century: An Introduction with Recommendations.. Science.

[12] Smith B, Ceusters W, Klagges B, Köhler J, Kumar A (2005). Relations in biomedical ontologies.. Genome Biology.

[13] Smith B (2004). The Logic of Biological Classification and the Foundations of Biomedical Ontology.. Medical Informatics.

[14] Masci AM, Arighi CN, Diehl AD, Lieberman AE, Mungall C (2009). An improved ontological representation of dendritic cells as a paradigm for all cell types.. BMC Bioinformatics.

[15] Mayr E (1982). The Growth of Biological Thought: Diversity, Evolution, and Inheritance..

[16] Valentine JW, May CL (1996). Hierarchies in biology and paleontology. .. Paleobiology.

[17] Mejino JLV, Agoncillo AV, Rickard KL, Rosse C (2003). Representing Complexity in Part-Whole Relationships within the Foundational Model of Anatomy.. In: Proceedings of AMIA Symp 2003.

[18] Kumar A, Smith B, Novotny DD (2004). Biomedical Informatics and Granularity.. Comparative and Functional Genomics.

[19] Vogt L (2010). Spatio-structural granularity of biological material entities.. BMC bioinformatics.

[20] Gupta A, Larson SD, Condit C, Gupta S, Fong L, Hainaut JL (2007). Toward an Ontological Database for Subcellular Neuroanatomy.. Lecture Notes in Computer Science.

[21] Wimsatt WC, Globus G, Maxwell G, Savodnik I (1976). Reductionism, Levels of Organization, and the Mind-Body Problem.. Consciousness and the Brain: A Scientific and Philosophical Inquiry.

[22] Wimsatt WC (1994). The Ontology of Complex Systems: Levels, Perspectives, and Causal Thickets.. Canadian Journal of Philosophy, Supplemental Volume.

[23] Salthe S (1985). Evolving Hierarchical Systems: Their Structure and Representation..

[24] Salthe SN (1993). Development and evolution: complexity and change in biology..

[25] Jagers Op Akkerhuis GAJM, van Straalen NM (1998). Operators, the Lego-bricks of nature, evolutionary transitions from fermions to neural networks.. World Futures The journal of general evolution.

[26] Riedl R (2000). Strukturen der Komplexität - Eine Morphologie des Erkennens und Erklärens..

[27] Keet CM (2008). A Formal Theory of Granularity - Toward enhancing biological and applied life sciences information system with granularity. Homepage of Maria Keet.. http://www.meteck.org/files/AFormalTheoryOfGranularity_CMK08.pdf.

[28] Eldredge N (1985). Unfinished Synthesis: Biological Hierarchies and Modern Evolutionary Thought..

[29] MacMahon JA, Phillips DL, Robinson JV, Schimpf DJ (1978). Levels of biological organization: an organism-centered approach.. BioScience.

[30] Levinton J (1988). Genetics, Paleontology and Macroevolution..

[31] Schulz S, Johansson I (2007). Continua in Biological Systems.. The Monist.

[32] Valentine JW (2004). On the origin of phyla..

[33] Jagers OP, Akkerhuis GAJM (2008). Analysing hierarchy in the organization of biological and physical systems.. Biological Reviews of the Cambridge Philosophical Society.

[34] Reitsma F, Bittner T, Kuhn W, Worboys M, Timpf S (2003). Scale in Object and Process Ontologies.. Spatial Information Theory.

[35] Bittner T, Smith B (2003). Granular Spatio-Temporal Ontologies.. AAAI Spring Symposium Papers.

[36] Rigaux P, Scholl M, Egenhofer M, H J (1995). Multi-scale partitions: Applications to spatial and statistical databases.. Proceedings Fourth International Symposium SSD95.

[37] Haendel MA, Neuhaus F, Osumi-Sutherland D, Mabee PM, Mejino JLV, Burger A, Davidson D, Baldock R (2008). CARO – The Common Anatomy Reference Ontology.. Anatomy Ontologies for Bioinformatics.

[38] Keet CM, Yao J (2010). A top-level categorization of types of granularity.. Novel Developments in Granular Computing: Applications for Advanced Human Reasoning and Soft Computation.

[39] Rector A, Rogers J, Bittner T (2006). Granularity, scale and collectivity: when size does and does not matter.. Journal of Biomedical Informatics.

[40] Bittner T (2004). Axioms for parthood and containment relations in bio-ontologies.. In: Proceedings of KR-MED 2004: First International Workshop on Formal Biomedical Knowledge Representation.

[41] Bittner T, Donnelly M (2007). A temporal mereology for distinguishing between integral objects and portions of stuff.. In:Proceedings of the Twenty-Second AAAI Conference on Artificial Intelligence, July 22-26, 2007, Vancouver, British Columbia.

[42] Rosse C, Mejino JL, Modayur BR, Jakobovits R, Hinshaw KP (1998). Motivation and Organizational Principles for Anatomical Knowledge Representation: The Digital Anatomist Symbolic Knowledge Base.. Journal of the American Medical Informatics Association.

[43] Rosse C, Mejino JLV, Burger A, Davidson D, Baldock R (2007). The Foundational Model of Anatomy Ontology.. Anatomy Ontologies for Bioinformatics: Principles and Practice.

[44] Krifka M, von Stechow A, Wunderlich D (1991). Massennomina.. Handbook of Semantics.

[45] Gillon BS (1992). Towards a Common Semantics for English Count and Mass Nouns.. Linguistics and Philosophy.

[46] Nicolas D (2008). Mass nouns and plural logic.. Linguistics and Philosophy.

