# Supplementary material for: Accommodating Ontologies to Biological Reality—Top-Level Categories of Cumulative-Constitutively Organized Material Entities
Source: PLoS One. 2012 Jan 9;7(1):e30004. doi: 10.1371/journal.pone.0030004 (PMC3253816; doi:10.1371/journal.pone.0030004)
Supplement: Table S1 — Definitions of additional Top-Level Categories of Material Entity required for Cumulative-Constitutively organized Material Entities. (DOC) [file pone.0030004.s001.doc]

## Table S1 - Definitions of additional Top-Level Categories of Material Entity required for Cumulative-Constitutively organized Material Entities

| **Definition** | **Parent Class Affiliation** |
| --- | --- |
| **'portion of matter':** *A material entity that is not demarcated by any physical discontinuities. At some finer level of granularity it is an object aggregate entity, at some coarser level of granularity it is a fiat object part entity, but at this level of granularity it is neither.*  *Examples: portion of blood plasma, portion of cytosol, portion of lymph plasma, portion of ECM, portion of cellular substance, portion of forest, portion of water, portion of sugar, portion of mortar, portion of dentin* | 'material entity' |
| **'portion of matter cluster':** *A material entity* [or *material entity cluster*] *that is a mereological sum of several clustered but spatially distinct portion of matter entities that are object aggregates at the same level of granularity. The portion of matter entities adhere to one another through chemical bonds or physical junctions that go beyond gravity. Examples: cluster of different types of portions of human tooth substance* | 'material entity' [*or* 'material entity cluster'] |
| **'portion of matter group':** *A material entity* [or *material entity group*] *that is a mereological sum of several scattered (i.e. spatially separated) portion of matter entities that are object aggregates at the same level of granularity and which do not adhere to one another through chemical bonds or physical junctions but relate to one another merely on grounds of metric proximity.  Examples: group of portions of dentine of an individual human being* | 'material entity' [*or* 'material entity group'] |
| **'portion of matter cluster group':** *A material entity* [or *material entity group*] *that is a mereological sum of several scattered (i.e. spatially separated) portion of matter cluster entities that are object aggregates at the same level of granularity. The portion of matter cluster entities do not adhere to one another through chemical bonds or physical junctions, but relate to one another merely on grounds of metric proximity.  Examples: group of clusters of different types of portions of human tooth substance from several teeth of an individual human being* | 'material entity' [*or* 'material entity group'] |
| **'granular mixture aggregate':** *A material entity that is a mereological sum of several separate material entities of different granularity levels and possesses non-connected boundaries.* | 'material entity' |
| **'object with portion of matter cluster':** *A granular mixture aggregate* [or *granular mixture cluster*] *that is a mereological sum of one object entity clustered with some portion of matter entity. The two entities adhere to one another through chemical bonds or physical junctions that go beyond gravity. Examples: single cell with surrounding ECM* | 'granular mixture aggregate' [*or* 'granular mixture cluster'] |
| **'object with portion of matter cluster group':** *A granular mixture aggregate* [or *granular mixture group*] *that is a mereological sum of several scattered (i.e. spatially separated) object with portion of matter cluster entities. The object with portion of matter cluster entities do not adhere to one another through chemical bonds or physical junctions, but relate to one another merely on grounds of metric proximity. Examples: group of spatially separated cells, each with its surrounding ECM* | 'granular mixture aggregate' [*or* 'granular mixture group'] |
| **'fiat object part with portion of matter cluster':** *A granular mixture aggregate* [or *granular mixture cluster*] *that is a mereological sum of one fiat object part entity clustered with some portion of matter entity. The two entities adhere to one another through chemical bonds or physical junctions that go beyond gravity. Examples: basal part of an epithelial cell with a portion of its basal lamina* | 'granular mixture aggregate' [*or* 'granular mixture cluster'] |
| **'fiat object part with portion of matter cluster group':** *A granular mixture aggregate* [or *granular mixture group*] *that is a mereological sum of several scattered (i.e. spatially separated) fiat object part with portion of matter cluster entities. The fiat object part with portion of matter cluster entities do not adhere to one another through chemical bonds or physical junctions, but relate to one another merely on grounds of metric proximity. Examples: a group of scattered basal parts of epithelial cells and their respective portions of basal lamina* | 'granular mixture aggregate' [*or* 'granular mixture group'] |
| **'object cluster with portion of matter cluster':** *A granular mixture aggregate* [or *granular mixture cluster*] *that is a mereological sum of one or more separate object cluster entities, all of which are clustered with the same portion of matter entity. The object cluster entities and portion of matter entity adhere to one another through chemical bonds or physical junctions that go beyond gravity. Examples: cell cluster with portion of ECM cluster* | 'granular mixture aggregate' [*or* 'granular mixture cluster'] |
| **'object cluster with portion of matter cluster group':** *A granular mixture aggregate* [or *granular mixture group*] *that is a mereological sum of several scattered (i.e. spatially separated) object cluster with portion of matter cluster entities. The object cluster with portion of matter cluster entities do not adhere to one another through chemical bonds or physical junctions, but relate to one another merely on grounds of metric proximity. Examples: a group of scattered cell clusters with their respective portions of surrounding ECM* | 'granular mixture aggregate' [*or* 'granular mixture group'] |
| **'object group with portion of matter cluster':** *A granular mixture aggregate* [or *granular mixture cluster*] *that is a mereological sum of several scattered (i.e. spatially separated) object entities, all of which are clustered with the same portion of matter entity. The object entities and portion of matter entity adhere to one another through chemical bonds or physical junctions that go beyond gravity. Examples: organelle group with portion of cytosol cluster, blood cell group with portion of blood plasma cluster, lymphocyte group with portion of lymph plasma cluster, brick group with portion of mortar cluster* | 'granular mixture aggregate' [*or* 'granular mixture cluster'] |
| **'object group with portion of matter cluster group':** *A granular mixture aggregate* [or *granular mixture group*] *that is a mereological sum of several scattered (i.e. spatially separated) object group with portion of matter cluster entities. The object group with portion of matter cluster entities do not adhere to one another through chemical bonds or physical junctions but relate to one another merely on grounds of metric proximity. Examples: several scattered cells at the organelle level of granularity, spatially separated portions of blood at the cellular level of granularity* | 'granular mixture aggregate' [*or* 'granular mixture group'] |
| **'fiat object part cluster with portion of matter cluster':** *A granular mixture aggregate* [or *granular mixture cluster*] *that is a mereological sum of one or more separate (i.e. not sharing a fiat boundary with each other) fiat object part cluster entities, all of which are clustered with the same portion of matter entity. The fiat object part cluster entities and portion of matter entity adhere to one another through chemical bonds or physical junctions that go beyond gravity. Examples: synapse, desmosome* | 'granular mixture aggregate' [*or* 'granular mixture cluster'] |
| **'fiat object part cluster with portion of matter cluster group':** *A granular mixture aggregate* [or *granular mixture group*] *that is a mereological sum of several scattered (i.e. spatially separated) fiat object part cluster with portion of matter cluster entities. The fiat object part cluster with portion of matter cluster entities do not adhere to one another through chemical bonds or physical junctions, but relate to one another merely on grounds of metric proximity. Examples: group of several synapses of an individual human being* | 'granular mixture aggregate' [*or* 'granular mixture group'] |
| **'fiat object part group with portion of matter cluster':** *A granular mixture aggregate* [or *granular mixture cluster*] *that is a mereological sum of one or more scattered (i.e. spatially separated) fiat object part group entities, all of which are clustered with the same portion of matter entity. The fiat object part group entities and the portion of matter entity adhere to one another through chemical bonds or physical junctions that go beyond gravity. Examples: group of blood vessels of a human lung clustered with the respective portion of surrounding ECM* | 'granular mixture aggregate' [*or* 'granular mixture cluster'] |
| **'fiat object part group with portion of matter cluster group':** *A granular mixture aggregate* [or *granular mixture group*] *that is a mereological sum of several scattered (i.e. spatially separated) fiat object part group with portion of matter cluster entities. The fiat object part group with portion of matter cluster entities do not adhere to one another through chemical bonds or physical junctions but relate to one another merely on grounds of metric proximity. Examples: groups of blood vessels of several organs of an individual human being clustered with their respective portions of surrounding ECM* | 'granular mixture aggregate' [*or* 'granular mixture group'] |
| **'object with fiat object part cluster with portion of matter cluster':** *A granular mixture aggregate* [or *granular mixture cluster*] *that is a mereological sum of one or more separate (i.e. not sharing a fiat boundary with each other) object with fiat object part cluster entities, all of which are clustered with the same portion of matter entity. The object with fiat object part cluster entities and the portion of matter entity adhere to one another through chemical bonds or physical junctions that go beyond gravity. Examples: human lung with its blood vessels and the respective portion of surrounding ECM* | 'granular mixture aggregate' [*or* 'granular mixture cluster'] |
| **'object with fiat object part cluster with portion of matter cluster group':** *A granular mixture aggregate* [or *granular mixture group*] *that is a mereological sum of several scattered (i.e. spatially separated) object with fiat object part cluster with portion of matter cluster entities. The object with fiat object part cluster with portion of matter cluster entities do not adhere to one another through chemical bonds or physical junctions, but relate to one another merely on grounds of metric proximity. Examples: a group of several human organs with their respective blood vessels and their respective portions of surrounding ECM* | 'granular mixture aggregate' [*or* 'granular mixture group'] |
| **'object with fiat object part group with portion of matter cluster':** *A granular mixture aggregate* [or *granular mixture cluster*] *that is a mereological sum of one or more separate (i.e. not sharing a fiat boundary with each other) object with fiat object part group entities, all of which are clustered with the same portion of matter entity. The object with fiat object part entities and the portion of matter entity adhere to one another through chemical bonds or physical junctions that go beyond gravity.* | 'granular mixture aggregate' [*or* 'granular mixture cluster'] |
| **'object with fiat object part group with portion of matter cluster group':** *A granular mixture aggregate* [or *granular mixture group*] *that is a mereological sum of several scattered (i.e. spatially separated) object with fiat object part group with portion of matter cluster entities. The object with fiat object part group with portion of matter cluster entities do not adhere to one another through chemical bonds or physical junctions but relate to one another merely on grounds of metric proximity.* | 'granular mixture aggregate' [*or* 'granular mixture group'] |
| **'portion of matter mixture cluster':** *A portion of matter mixture aggregate* [or *granular mixture cluster*] *that is a mereological sum of several portion of matter entities that are object aggregates at different levels of granularity (forming a granular mixture). The portion of matter entities adhere to one another through chemical bonds or physical junctions that go beyond gravity. Examples: portion of blood, portion of lymph, portion of tissue, portion of brick wall* | 'portion of matter mixture aggregate' [*or* 'granular mixture cluster'] |
| **'portion of matter mixture cluster group':** *A portion of matter mixture aggregate* [or *granular mixture group*] *that is a mereological sum of several scattered (i.e. spatially separated) portion of matter mixture cluster entities. The portion of matter mixture cluster entities do not adhere to one another through chemical bonds or physical junctions but relate to one another merely on grounds of metric proximity. Examples: spatially separated portions of blood* | 'portion of matter mixture aggregate' [*or* 'granular mixture group'] |
| **'portion of matter mixture group':** *A portion of matter mixture aggregate* [or *granular mixture group*] *that is a mereological sum of several scattered (i.e. spatially separated) portion of matter entities that are object aggregates at different levels of granularity (forming a granular mixture), which do not adhere to one another through chemical bonds or physical junctions but relate to one another merely on grounds of metric proximity.* | 'portion of matter mixture aggregate' [*or* 'granular mixture group'] |
| **'material entity aggregate':** *A material entity that is a mereological sum of several separate material entities and possesses non-connected boundaries.* | 'material entity' |
| **'material entity cluster':** *A material entity aggregate that is a mereological sum of several separate material entities, which adhere to one another through chemical bonds or physical junctions that go beyond gravity.* | 'material entity aggregate' |
| **'material entity group':** *A material entity aggregate that is a mereological sum of several scattered (i.e. spatially separated) material entities, which do not adhere to one another through chemical bonds or physical junctions but, instead, relate to one another merely on grounds of metric proximity. The material entities are separated from one another through space or through other material entities that do not belong to the group.* | 'material entity aggregate' |
| **'granular mixture cluster':** *A material entity cluster that is a mereological sum of several separate material entities of different granularity levels, which adhere to one another through chemical bonds or physical junctions that go beyond gravity.* | 'material entity cluster' |
| **'granular mixture group':** *A material entity group that is a mereological sum of several scattered (i.e. spatially separated) material entities of different granularity levels, which do not adhere to one another through chemical bonds or physical junctions but, instead, relate to one another merely on grounds of metric proximity. The material entities are separated from one another through space or through other material entities that do not belong to the group.* | 'material entity group' |
